# Evidence for multiple past introgressions among chimpanzee subspecies during warming periods

**DOI:** 10.1038/s42003-026-10437-z

**Published:** 2026-06-05

**Authors:** Alessandro Mondanaro, Marina Melchionna, Carmela Serio, Giorgia Girardi, Axel Timmermann, Elke Zeller, Silvia Castiglione, Alessia Di Costanzo, Linda Morvillo, Antonella Esposito, Alessandra de Durante, Mirko Di Febbraro, Pasquale Raia

**Affiliations:** 1 DiSTAR, University of Naples Federico II, Naples, Italy; 2 IBS Center for Climate Physics, Institute for Basic Science, Busan, South Korea; 3 Department of Geosciences, University of Arizona, Tucson, AZ USA; 4 EnviXLab, Department of Biosciences and Territory, University of Molise, Pesche (Isernia), Italy

**Keywords:** Evolution, Ecology

## Abstract

The evolutionary history of common chimpanzee (*Pan troglodytes*) subspecies is complex, marked by several past introgression events whose dating and location are unknown and are at odds with the current strong geographic segregation of the subspecies. We reconstruct the historical biogeography of common chimpanzee subspecies. Our findings reveal that cranial morphology reflects genetic differentiation, with the western chimpanzee being the most derived lineage. This is consistent with its current geographic isolation, which we find was punctuated by periods of potential contact with the other subspecies. These moments coincide with warming periods creating increased tree cover across the geographic barrier, the Dahomey Gap, currently separating western chimpanzee from the other subspecies. This integrative framework of a climate-mediated dynamical biogeography provides unprecedented insights into the dynamics of chimpanzee evolution, demonstrating how past climatic fluctuations and vegetation changes profoundly shaped the present-day distribution of *Pan troglodytes subspecies* along with their genetic differences.

**Figure:**
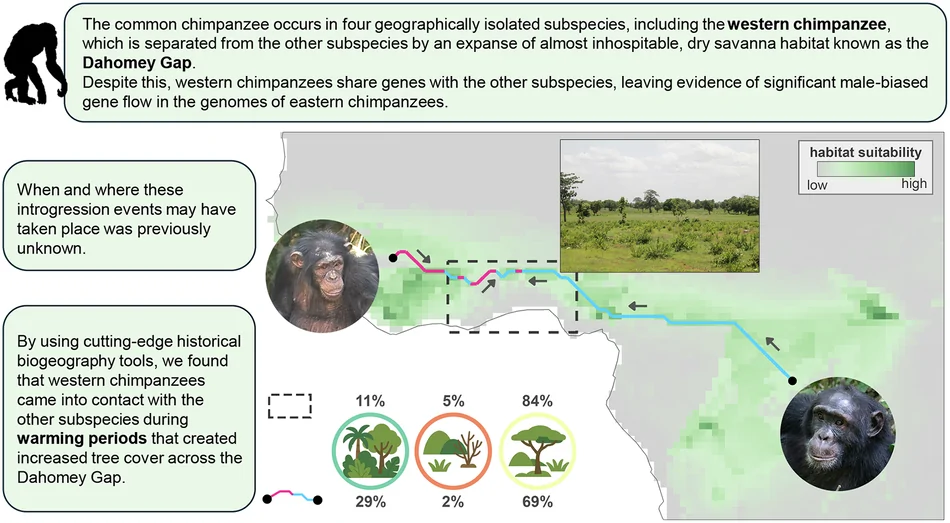

## Abstract (web summary)

Despite living isolated by the other subspecies, today’s western chimpanzees share genes with them. Historical biogeography reveals gene flow likely occurred during ancient warming periods, when expanded tree cover successfully bridged the vast gap.

## Introduction

Chimpanzees are humans’ closest living relatives^1^. The genus *Pan* comprises two extant species, the common chimpanzee (*Pan troglodytes*) and the bonobo (*Pan paniscus*). The common chimpanzee includes four genetically and geographically separate subspecies, reflected in distinctive behavioural attributes, ecological characteristics, morphology, and selection for alleles conferring specific adaptations to their respective habitats^1–7^.

**Fig. 1:**
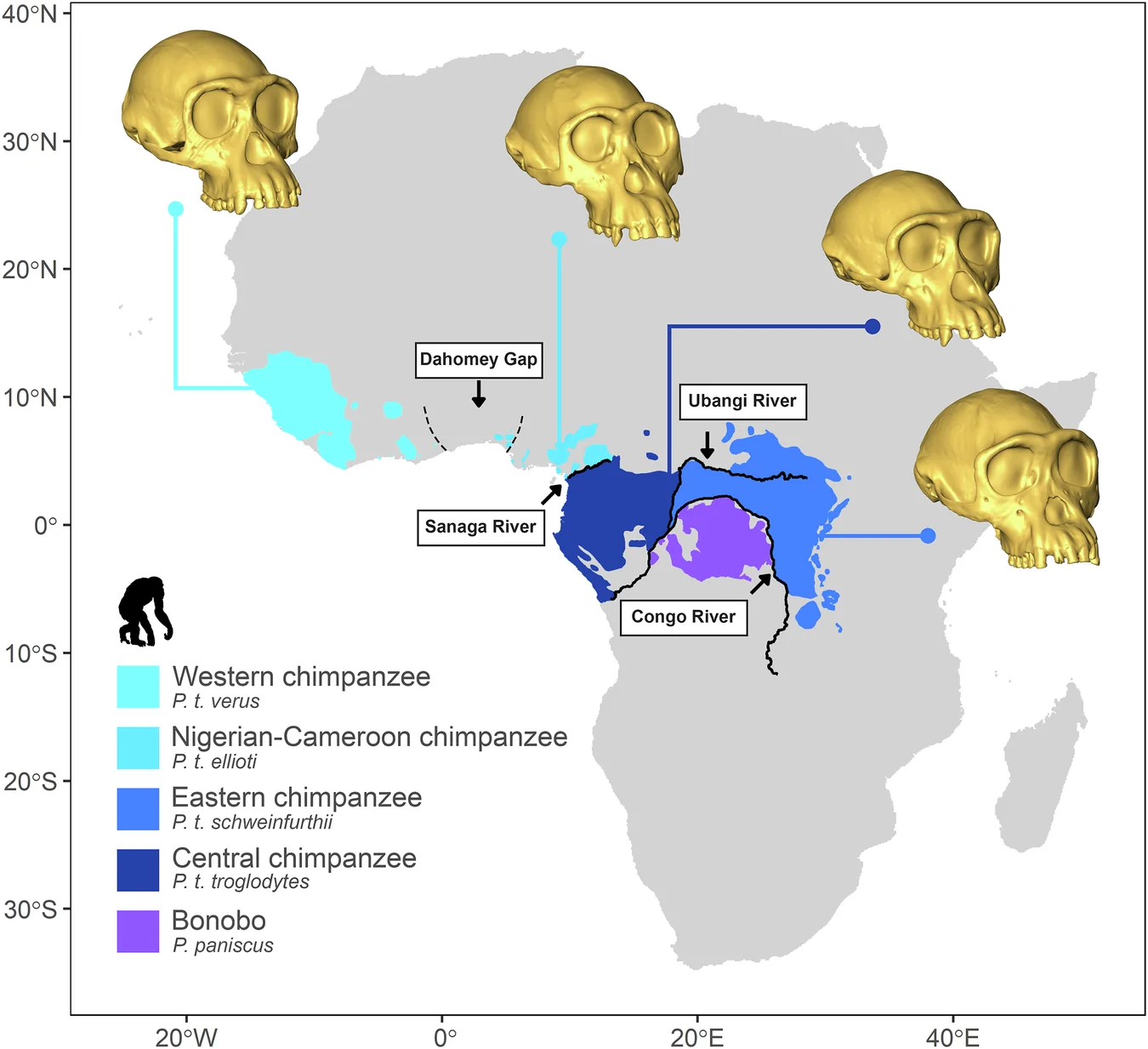
Geographical distribution of the *Pan* genus. Spatial polygons of *Pan* genus derived from the IUCN Red List and the cranial morphology associated to each subspecies.

The four subspecies are separated from each other by little-permeable ecological barriers such as the Sanaga and the Ubangi rivers and from bonobos by the Congo River. *P. t. ellioti* (the Nigeria-Cameroon or NC chimpanzee), occurs north and west of the Sanaga River. *P. t. troglodytes* (the central chimpanzee) inhabits the Guinean-Congolian lowland forests and swamp forests south to the Sanaga^7^. *P. t. schweinfurthii* (the eastern chimpanzee) occurs in lowland and montane forests, and forest-savanna mosaics from the northern Democratic Republic of the Congo eastward^1,8^. Finally, the western chimpanzee (*P. t. verus*) is further disconnected by the other three common chimpanzee subspecies by an expanse of inhospitable, dry savannah habitat known as the Dahomey Gap, running along the West African coastline through Togo, Benin and Ghana^9^ (Fig. 1).

The geographic segregation between common chimpanzee subspecies is mirrored by profound biological differentiation, particularly in the western chimpanzee. Based on morphology, mandible and molar shapes consistently differentiate this subspecies from the others^5,10,11^. Behaviourally, it stands out for the cultural transmission of unique traits^12–14^, including tool-crafting for hunting, high rate of meat consumption, and, uniquely, both unusually strong social bonding and high philopatry among females^15–18^. These traits underscore a deep divergence consistent with its remote geographical position and genetic distinctiveness^4,19–21^.

Brand et al.^22^ argued that the split between the common chimpanzee and the bonobo occurred approximately around 2 Ma and that the most recent common ancestor to the four extant common chimpanzee lineages lived some 1 Ma. De Manuel et al.^23^ estimated the age of the split between a western plus NC chimpanzee clade and the central plus eastern chimpanzee clade at some 600 ka. The subsequent splits between western and NC chimpanzees, and between the central and eastern chimpanzee, were estimated at around 250 ka and 150 ka, respectively.

Although the phylogenetic relationships are robust and divergence times among members of the common chimpanzee clade are distant in time, the evolutionary history of its subspecies is still poorly understood^22,24,25^, hindered by the virtual absence of a fossil record^26^, currently limited to approximately 500 ka old fossil teeth found in Kapthurin Formation, west of Lake Baringo in Kenya^27^, and complicated by multiple introgression events between them, whose dating remains almost unknown^19,23,28–31^. Surprisingly, it was found that up to 21% of autosomal DNA in the eastern chimpanzee genome derives from male-biased western chimpanzee introgressions^22^, consistent with migration models showing unidirectional immigration from western into both eastern and central chimpanzees and their ancestor^28,30,31^. This in turn suggests that either the Dahomey Gap was intermittently permeable to either western or eastern chimpanzees^9,32,33^ or that they met within the Gap at some point, in keeping with their proclivity to live in relatively dry, savannah habitats^34–36^.

These alternatives are difficult to evaluate since the Dahomey Gap has undergone periods of expansion, contraction, and vegetational cover change^9,32,33^ potentially creating intermittent corridors between western and central/eastern/NC chimpanzee populations, which are not currently reconciled with any known instance of introgression. Furthermore, significant variations in effective population size of individual subspecies through time^19,22,28,29^, the persistence of glacial refugia within the actual range of the subspecies^37^, and improved behavioural plasticity in chimpanzee lineages in response to variable environmental conditions^2^ (arguably conferring on some *P. troglodytes* populations a greater capacity for migration than others), further complicate the issue. Whatever the reason, the dating of the western to eastern chimpanzee introgression event, estimated by Brand et al.^22^ at approximately 16.7 ka, is associated with considerable uncertainty, to the extent that an older, albeit unknown, estimate is suggested by the same authors^22^. Traces of several other introgression events are found within *P. troglodytes* genome, but their dating is even more uncertain than in the western to eastern chimpanzee gene flow case, nor is it clear whether these events pertain to the currently living subspecies, or to any older, undifferentiated lineage^19,22,23,28–31^. In fact, a zone of hybridisation may have existed between NC and central chimpanzees in Cameroon^38^, and further introgression certainly occurred between NC, central and eastern chimpanzees^19,31,39^, between western and central chimpanzees^22^ and even between bonobos and non-western chimpanzees^19,23^. The intricate introgression history, covered by layers of temporal uncertainty, is in stark contrast with the geographically clear-cut geographic separation of the extant common chimpanzee subspecies, leaving unanswered the question of how, where and when the subspecies came in contact. This is particularly true of the introgression events that occurred through the Dahomey Gap, which currently sets the western chimpanzee apart from any other subspecies. Here, we tackle these issues by integrating morphological, ecological, and evolutionary analyses to reconstruct the biogeographic history of *Pan troglodytes*. We address the contrast between the western chimpanzee’s distinctiveness and its genetic connectivity by testing two specific hypotheses regarding the mechanisms of divergence and dispersal.

First, leveraging previous, albeit limited, evidence^5,10,11^, we hypothesise that the geographic isolation of the western chimpanzee has driven profound phenotypic differentiation, making it the most morphologically derived subspecies. This serves to quantify the effectiveness of the Dahomey Gap as a long-term obstruction to gene flow. Secondly, we hypothesise that climatic oscillations modulated the effectiveness of Dahomey as an ecological barrier. We predict that specific interglacial intervals increased tree cover sufficiently to create temporary “permeability windows” facilitating range expansions and providing the potential for contact between western chimpanzees and other subspecies.

To test these hypotheses, we applied phylogenetic comparative methods to high-resolution 3D cranial data to quantify the direction and magnitude of shape evolution. We then employed a recently developed method to infer the area of origin (AOO) of all extant *Pan* lineages. Finally, we modelled the spatial environmental corridors of the western chimpanzee migrations over the last 250 ka to assess the potential for gene flow and admixture between western chimpanzee and the other *P. troglodytes* subspecies (Materials and Methods, Supplementary Information). This integrative framework provides critical insights into how geographic and environmental processes have shaped the present-day distribution and genetic structure of our closest extant relatives.

## Results

### The shape and the direction of the morphological differentiation among *Pan troglodytes* subspecies

**Fig. 2:**
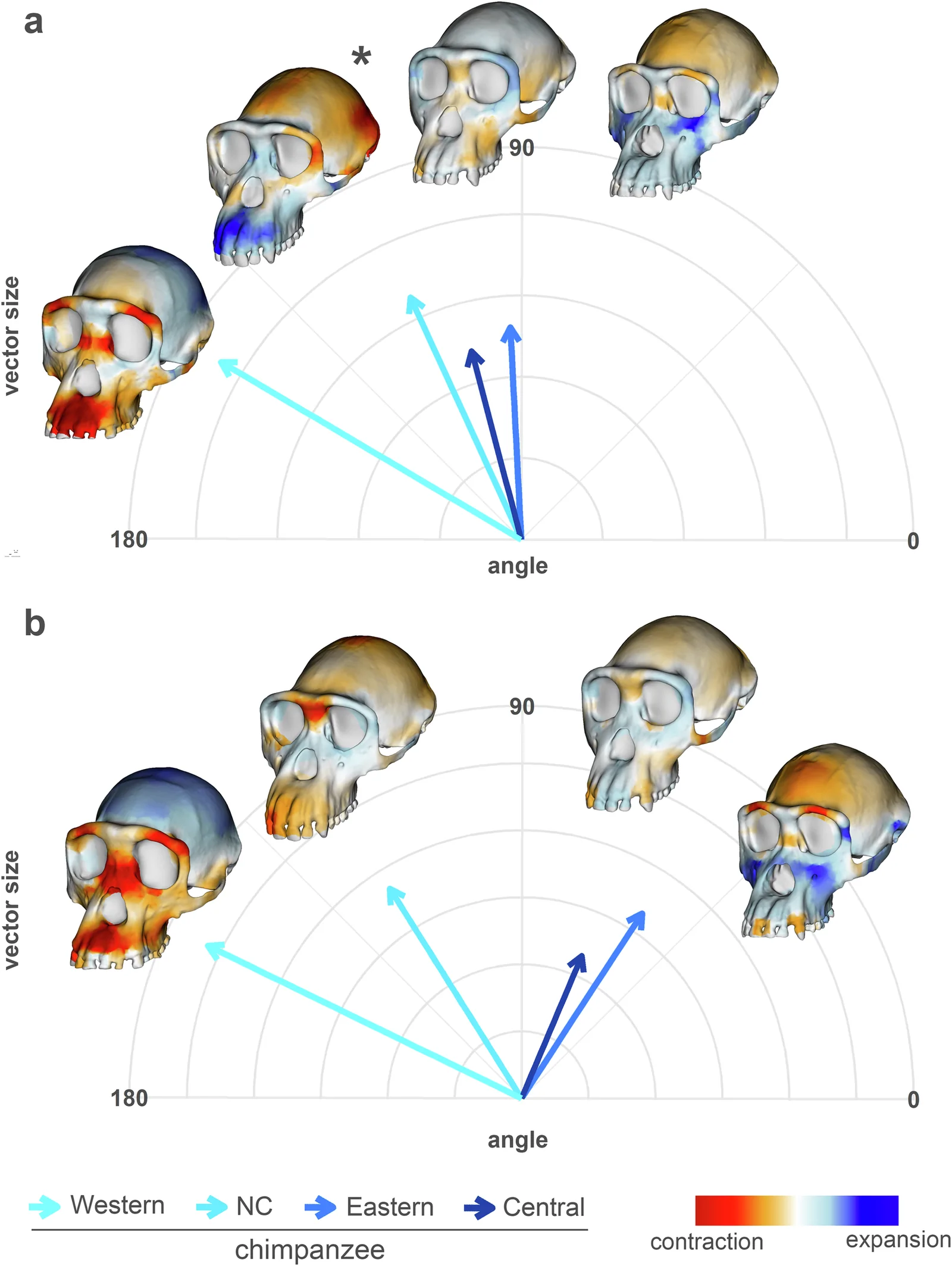
Direction and magnitude of the morphological change among *Pan troglodytes subspecies.* *Pan* crania were coloured according to the areas that were under highest evolutionary rates in terms of expansion (blue areas) and contraction (red areas) from the most recent common ancestor (MRCA) estimated morphology. The multivariate angles were computed between each subspecies’ and their estimated MRCA shapes^40^. Vector length (magnitude) is proportional to the total shape transformation from the ancestor to the extant subspecies. The angle describes the direction of phenotypic drift in the multivariate space. The distinct orientation and greater length of the western chimpanzee vector (cyan) quantitatively demonstrate that it is the most morphologically derived subspecies. **a** refers to a combined dataset of males and females. **b** repeats the analysis using females only to account for the absence of male Nigeria-Cameroon (NC) chimpanzee specimens.

In terms of direction and magnitude of evolutionary shape change, we found that the western chimpanzee forms the morphologically most derived subspecies (Fig. 2, Supplementary Figs. 1–3). The directions of evolutionary shape changes replicate the phylogenetic position of the subspecies, indicating that morphological and genetic information are consistent. Repeating the analysis using only female data (as we miss male representatives of NC chimpanzees) provides qualitatively the same results (Fig. 2b).

### Areas of origin of *Pan* lineages

**Fig. 3:**
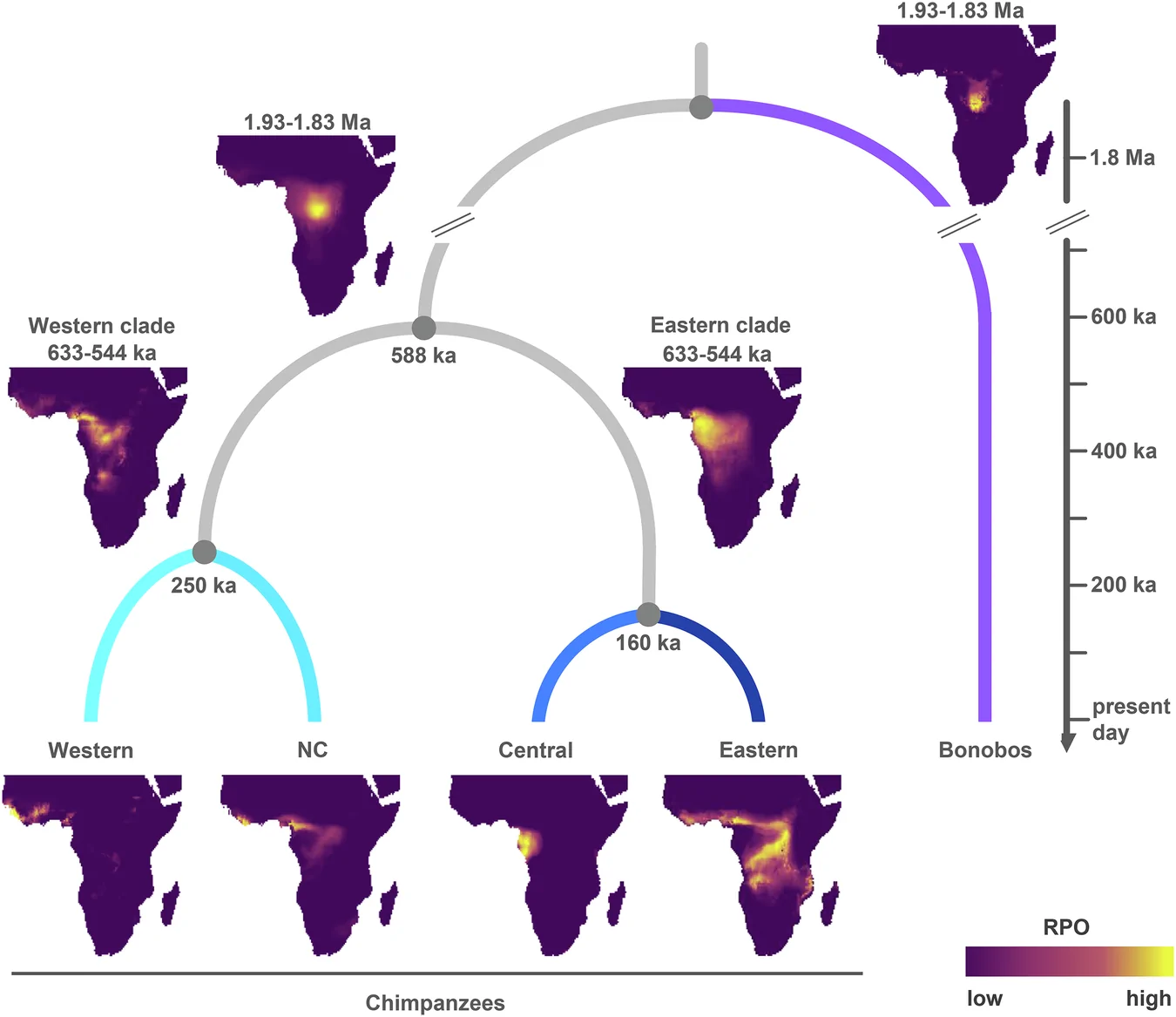
Phylogenetic position and RRphylogeography maps of *Pan* lineages. Each map represents the estimated relative probability of occurrence (RPO) of the area of origin for each lineage.

To calculate the AOO, we first trained species distribution models for each subspecies based on MaxEnt^40^ algorithm and investigated the habitat suitability distributions during hypothesised divergence times (see Materials and Methods for more details) using the *RRphylogeography* method^41^. The AOO of individual *Pan* lineages mostly reflects their current geographic distribution^7,19,21^. This is true both for the two nominal *Pan* species, and for western and eastern clades and individual subspecies within these clades (Fig. 3).

Unexpectedly, the AOO of NC chimpanzee extends beyond its current range to include an additional region to the west, roughly coinciding with the Upper Guinean Rainforest (Fig. 3).

### Migration Pathways between *Pan troglodytes* subspecies

**Fig. 4:**
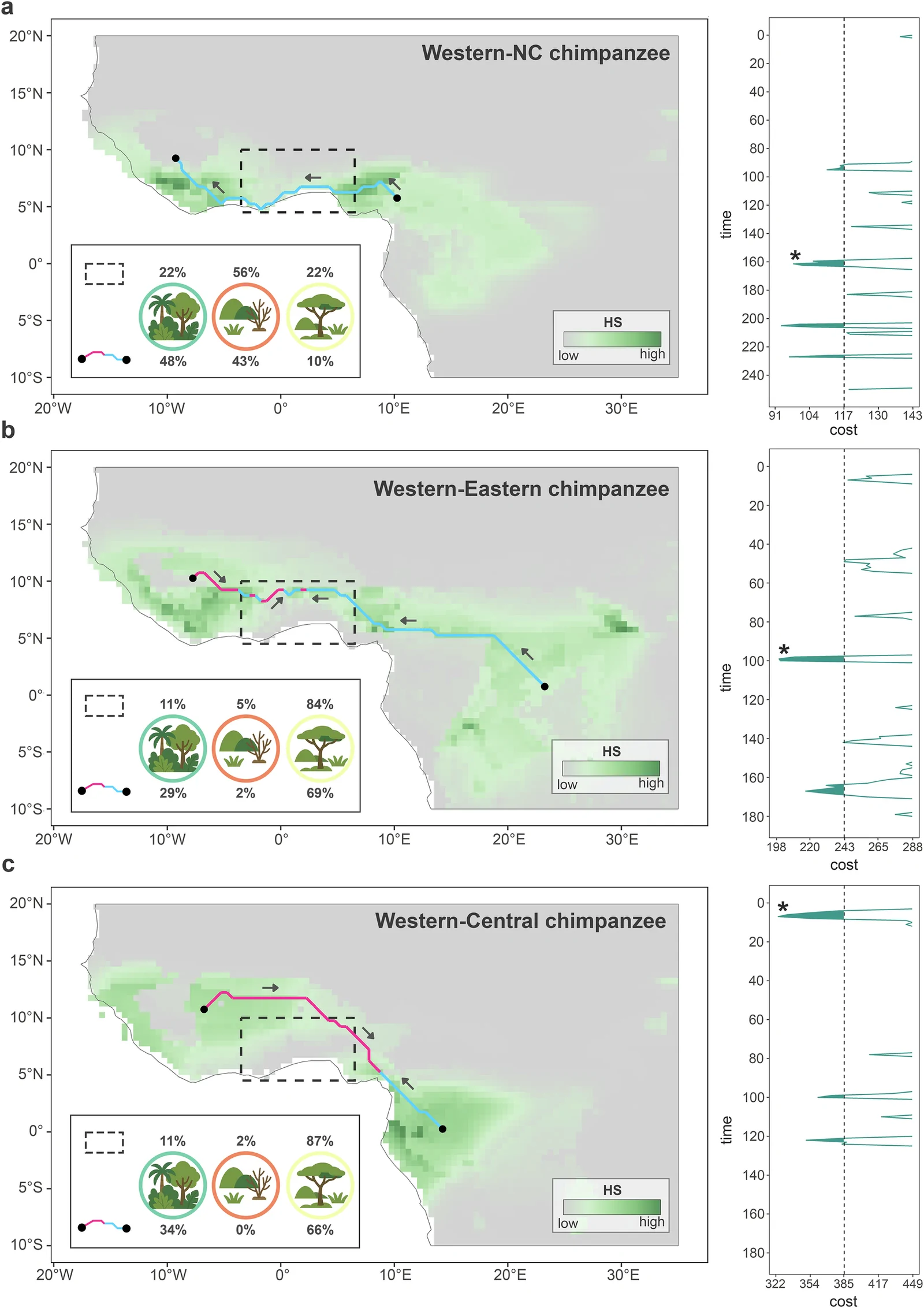
Combined habitat suitability maps (HS, green palette) for pairs of common chimpanzee subspecies at the moment of the highest probability of contact. The timing of contact is indicated by an asterisk – **a** 163 ka, **b** 99 ka, **c** 7 ka - along the curve charting the lowest cost for migration from one population to the other reported in the right column). For each pair, we also identified the least cost path from the barycentre (the geographic mean weighted by habitat suitability values, Materials and Methods) of individual populations. Segments along the path are coloured according to the subspecies possessing the highest habitat suitability value within the pair (deep pink always refers to western chimpanzee, light blue to the other subspecies of the pair). The circle insets show the biomes in the boxed region where green, orange and yellow correspond to tropical forest, savannah and grassland megabiomes, respectively. The percentages above the circles represent the percentage of each megabiome type within the dashed box area. The percentages below the circles represent the percentage of each megabiome type along the path connecting the two subspecies barycenters.

To better understand the dynamics of divergence and introgression, we identified several potential migration routes pairing the western chimpanzee to the other subspecies over the past 250,000 years (Fig. 4), indicating that potential contact between the subspecies occurred during periods of relative warming (Supplementary Fig. 4).

We found four optimal time-windows pairing western and NC chimpanzees (Fig. 4a, right panel, Supplementary Fig. 5, Supplementary Tables 3, 4). The two oldest occur close to the presumed age of the most recent common ancestor to these subspecies, during Marine Isotope Stage 7 (MIS 7, an interglacial period). A third time window occurs in between 167 and 160 ka, topping at 163 ka, during a transient warm spell through MIS 6 glacial^42^ (Supplementary Fig. 4). The last possible, although much less probable, migration window occurs through the 95 to 92 ka interval, during MIS 5 interglacial (Supplementary Fig. 5b).

**Fig. 5:**
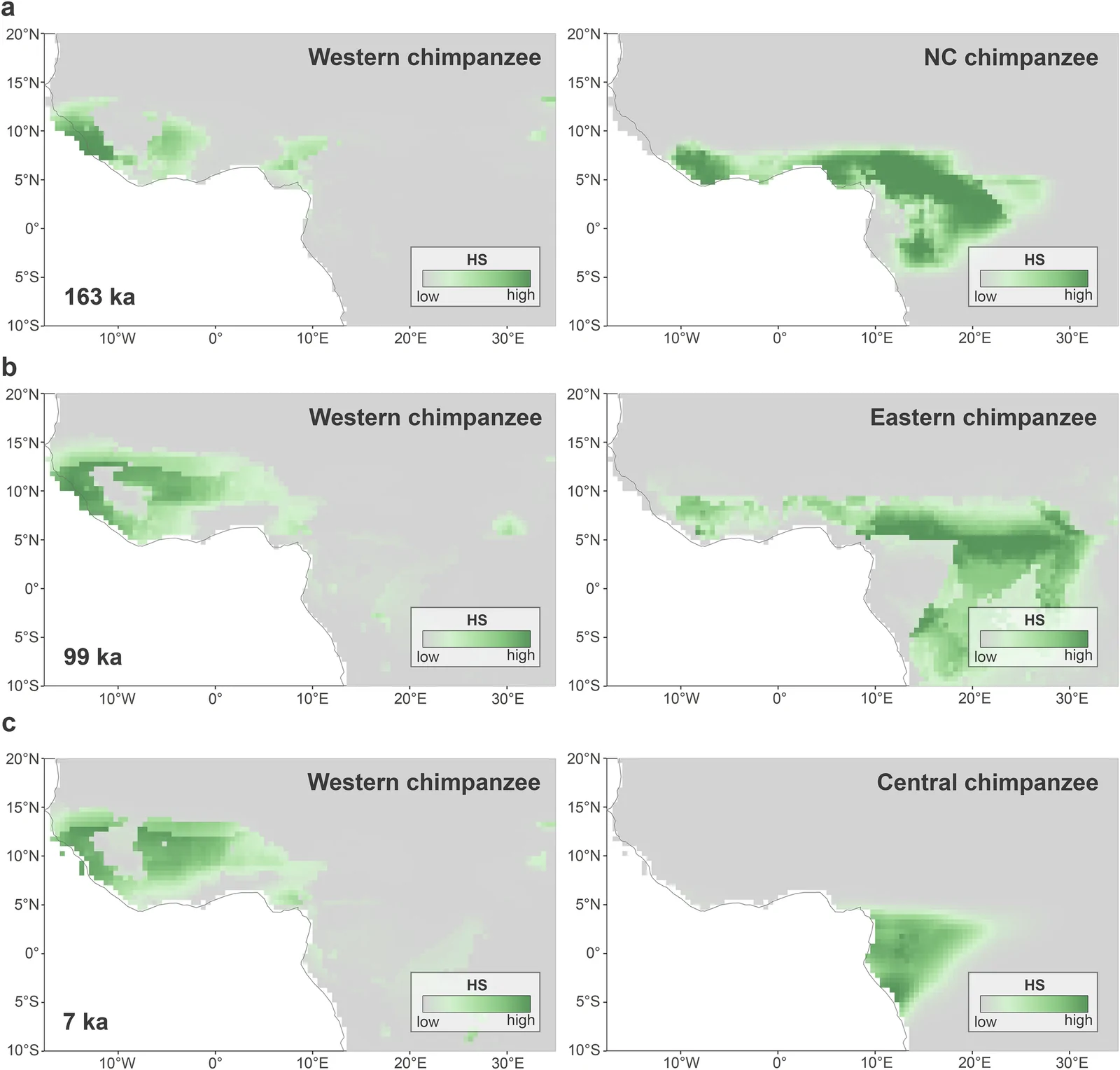
Habitat suitability maps for individual subspecies at the moment of the highest probability of contact between each pair. **a** Western-NC chimpanzees contact at 163 ka; **b** Western-Eastern chimpanzees contact at 99 ka; **c** Western-Central chimpanzees contact at 7 ka. The colour bar is the same scale as in Fig. 4.

At 163 ka, the habitat suitability maps of NC chimpanzee extend west of the Dahomey Gap (Fig. 5a), whereas the suitability of the western chimpanzee is high just in the Upper Guinean Rainforest, where the species lives today (and where it found refugium during glacial times^37^), suggesting the NC chimpanzee individuals reach out to the western population rather than the other way around. Using BIOME4 paleovegetation simulations conducted with BIOME4^43^, we found that the tropical forest megabiome^44^ covered some 21.8% of the area including the Dahomey Gap (Fig. 4a) at 163 ka, compared to 17% today and as low as 6% during the last glacial maximum at 22 ka (Supplementary Table 3). A mosaic of tropical forests and savannah runs parallel to the coastline and through the Gap (Fig. 4a). This represents the preferred environment of NC chimpanzee^7,45^ and the path with the highest conductance map connecting the barycentre of the two subspecies suitability maps at 163 ka. Accordingly, it is reasonable to assume that NC chimpanzee crossed the Dahomey Gap at around 163 ka in its migration path toward western chimpanzee territory (Fig. 4a). In keeping with this assumption, we found that the 47.5% of path was covered by tropical forests at that time (Fig. 4a, Supplementary Table 4). Since this is more than twice the coverage of tropical forests present in the Gap area at this time, this is a clear indication that migrating NC chimpanzees selected this habitat type through the vegetation mosaic connecting the two subspecies. Repeating the same procedure during the 95 to 92 ka interval strengthened this pattern. The migration cost is lowest at 95 ka, when savannah and tropical forest collectively covered 71% of the reference area, 79.5% along the path with three times as much tropical forest cover as in the Dahomey area (Supplementary Tables 3, 4).

The ideal time windows for contact between western and eastern chimpanzee occur during almost the same intervals as the western and NC chimpanzee duo, topping at 99 ka (Fig. 4b, right panel, Supplementary Fig. 6, Supplementary Tables 3, 4). Differing from the latter, though, these potential migration routes do not run through, but somewhat north of the Dahomey Gap. The habitat suitability maps of the two subspecies at 99 ka do not suggest either one ventured into the other’s range (Fig. 5b). Conversely, both exhibit high suitability values along the highest conductance (i.e. lowest cost) path, suggesting a possible case for introgression driven by range expansion of both subspecies. Vegetation cover at that time suggests a strong preponderance of the savannah megabiome, covering as much as 84% of the total reference area, but much higher forest cover along the path (29% compared to 11% within the reference area (Fig. 4b, Supplementary Tables 3, 4). This indicates the moment was ideal for the two subspecies to come in contact, in keeping with their ability to fare well in savannah environments even today^34–36^.

Western and central chimpanzee first possibly came in contact during the Eemian period (around 122 ka), and around 100 ka (both within MIS 5), always north of the Dahomey Gap (Fig. 4c, Supplementary Fig. 7). The last and best time window, though, occurs during the Holocene around 7 ka (Fig. 4c). Habitat suitability maps suggest western chimpanzee likely ventured eastward of its current range (Fig. 5) taking advantage of the dominance of savannah within and around the Dahomey Gap (covering 87% of the area, Fig. 4c, Supplementary Table 3). Along the reconstructed path, though, tropical forests are again better represented than in the reference area (34%, Supplementary Table 4).

## Discussion

The current distribution of *Pan troglodytes* subspecies is governed by geographic barriers and conditioned by the chimpanzees’ notorious reluctance to cross waterways^7,46^ (Fig. 1). This long-standing geographic segregation has shaped the phylogeography of the genus, limiting gene flow between distant populations and favouring deep biological differentiation across subspecies^19,20,46,47^, cultural diversification across space^2,12–14,48^, and igniting adaptation to local conditions which span from resistance to malaria in forest chimpanzees to resistance to Simian Immunodeficiency Virus (SIV) in the savannah chimpanzee^4,39^. On the morphological grounds, our analyses demonstrate that although western chimpanzee is morphologically closest to its nearest relative, the NC chimpanzee, pointing to a sizeable effect of phylogenetic inheritance, it is the most distinct of all *P. troglodytes* subspecies (Fig. 2, Supplementary Fig. 3). Juvenile western chimpanzees exhibit a characteristic dark “mask” over the nose and eyes, contrasting with the otherwise pink coloration of the face. Traces of this mask are usually still visible in adulthood. In contrast, juveniles of the other subspecies typically have uniformly pink faces, which may later develop large tan spots that eventually coalesce^49^. During adulthood, *P. t. verus* develops a full, rounded white chin beard, in contrast to the sparser white pelage observed over the other subspecies' chin. Additional morphometric differences have been documented in cranial anatomy^6,10,11^. To the latter we add here that western chimpanzee has narrower muzzle and supraorbital torus, and more rounded, less flattened neurocranium relative to the other subspecies (Fig. 2, Supplementary Fig. 2). The magnitude of this phenotypic shift confirms that the Dahomey Gap has acted as an exceptionally effective barrier, allowing western chimpanzee to undergo significant evolutionary drift in near-total isolation from its counterparts east of the Gap, conferring it its distinctive genome^4,19,20^, and behaviour^1,12,16,18^. And yet, this isolation was punctuated by moments when the western chimpanzee exchanged genes with the other subspecies in the past, although when, and where this genetic admixture took place is currently unknown^22^. By using a recently developed, accurate historical biogeography tool^41^, coupled with a specific approach sought to estimate the best (most suitable) migration routes between any pair of lineages. We demonstrated that there were at least two different windows of time when migration from and to western chimpanzee habitats in the Upper Guinean Forests and surrounding savannah environments might have occurred. The first, during the 167 to 160 ka time window, was most profitable for contact between the western and NC chimpanzee. At 163 ka in particular, tropical forest and savannah environments each covered 21.8% of the area engulfing the Dahomey Gap. In comparison, in preindustrial times, the Gap is 81.6% savannah and dry woodland, 1.2% grassland and dry shrubland and 17.2% tropical forest. As a reference figure, during the Last Glacial Maximum (LGM), tropical forest and savannah megabiomes covered collectively as little as 6.7% of the area (Supplementary Table 3). The greater extent of tropical forest coverage at 163 ka was ideal for NC chimpanzee to extend its range westwards as to reach the territory suitable for the western chimpanzee, as confirmed by the fact that the path connecting NC to the western chimpanzee runs through an even greater proportion of tropical forest megabiome (Supplementary Table 4). The western (along with the eastern) chimpanzee is often referred to as the ‘savannah’ chimpanzee^36^ for its ability to thrive in open woodlands, savannah-like environments. High seasonality in food availability may also occur in more forested habitats, as it is certainly the case for NC chimpanzee^50^. This implies that a Dahomey corridor may have formed and become permeable to NC but not to western chimpanzee, provided more humid conditions favoured higher tree density. On interannual timescales, the Dahomey Gap receives high rainfall when the equatorial Atlantic upwelling collapses, such as during Atlantic Niño events (Supplementary Fig. 9). On orbital timescales, colder glacial sea surface temperatures in the equatorial Atlantic, as recorded e.g., in marine sediment cores, would also favour drier conditions, a pattern that reverts during interglacials^51^. This effect can be furthermore modulated by the precessional cycle, which changes the meridional temperature gradient and hence the movement and extent of the Western African Monsoon front. Consistent with this mechanism, the Dahomey Gap became forested multiple times during the last 520 ka, coinciding with interglacial periods when more humid conditions dominate^33^. Similarly, the AOO of NC and western chimpanzee partially coincided with MIS 7, which is yet another interglacial concurrent with one of the six Dahomey Gap foresting episodes^33^. Intriguingly, the 167 to 160 ka interval also included an intermediate warm episode within MIS 6 d, to the extent that a marine transgression in Namaqualand (southern Africa) brought sea levels to modern heights^42^.

All this evidence strengthens the notion that a moment of population overlap may have occurred around 163 ka, thanks to NC chimpanzee traversing a stretch of mosaic environments running parallel to the coastline (Fig. 4a).

During the stretch of time extending from 100 to 95 ka, that is well within the MIS 5 interglacial, further contact zones may have formed between the western chimpanzee and all other subspecies, and towards the eastern chimpanzee in particular. The great extension of the savannah at 99 ka indicates that both subspecies may have traversed a narrow strip north of the Dahomey Gap, dominated by a savannah environment^34–36^ at that time (Fig. 4b). Genetic evidence points to western chimpanzee male genome introgression into the eastern subpecies^22^. This may have been favoured by higher gregariousness and philopatry in western chimpanzee females^52–54^, assuming that it was easier for female eastern chimpanzee to mate with male western chimpanzee than the other way around, as the two expanded towards the Gap.

**Fig. 6:**
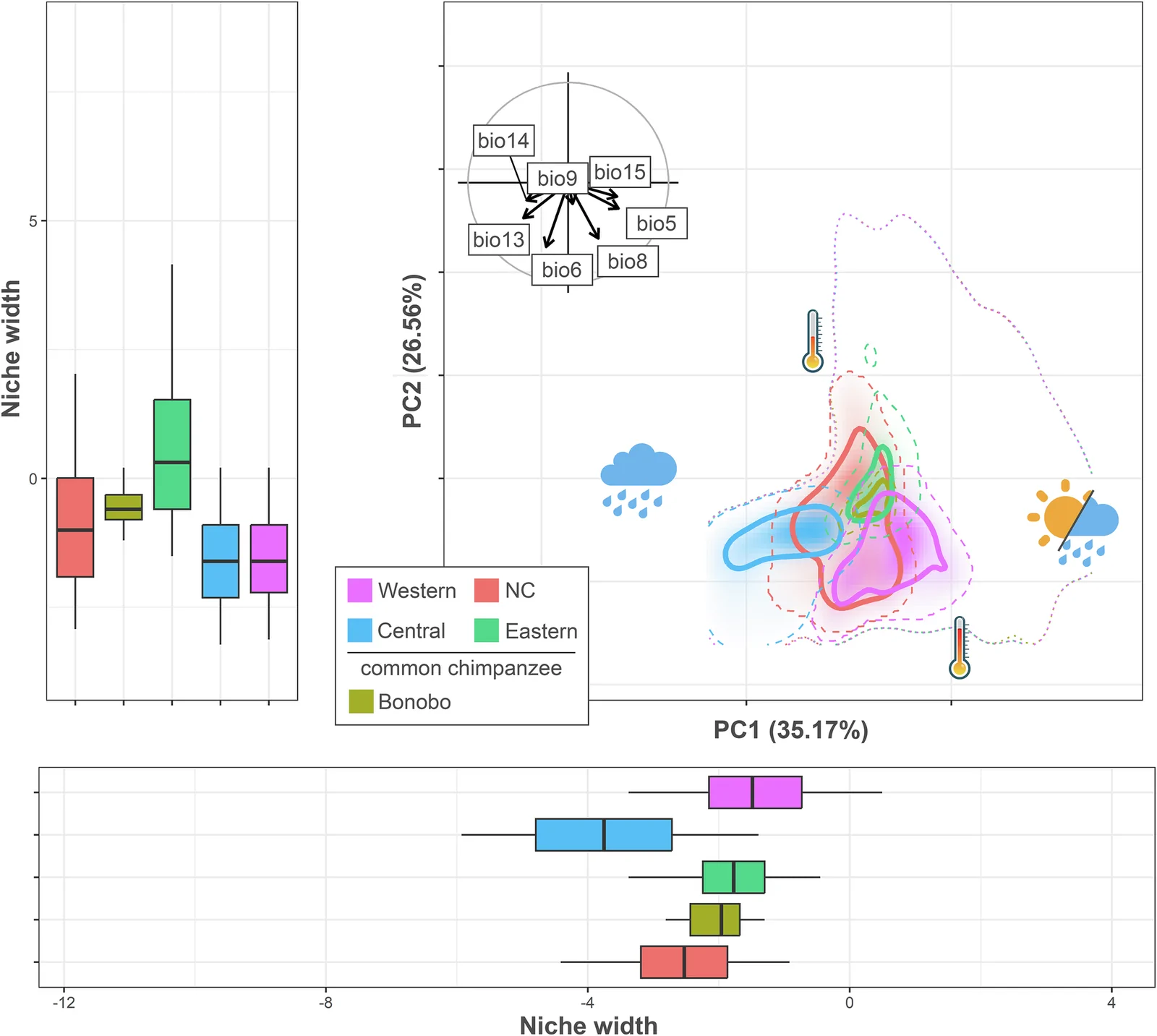
Niche positions and differences between *P. troglodytes* subspecies. Estimated niche probability of 4 chimpanzee subspecies in the PC1-PC2 state space spanned by the climatic vector components: bio4 (temperature seasonality), bio5 (max temperature of warmest month), bio6 (min temperature of coldest Month), bio8 (mean temperature of the wettest quarter), bio9 (mean temperature of driest quarter), bio13 (precipitation of wettest month), bio14 (precipitation of driest month), and bio15 (precipitation seasonality). Solid lines enclose 50% of the climatic niche space for each *Pan* subspecies, while dashed lines encompass 90% of their niche space. Dotted line encloses 95% of the background climatic conditions (i.e., climatic variation across the study area).

Although we found the 100 to 95 ka interval was profitable for contact between western and any other subspecies, the central chimpanzee’s high affinity to closed forest environments makes yet another interval, at 7 ka, the most likely one for the two species to meet (Fig. 4c). At that time, the area engulfing Dahomey was some 88% savannah, meaning there is little chance for the central chimpanzee to traverse it. However, we found that the western chimpanzee was able to migrate north of Dahomey Gap to reach the central chimpanzee’s territory. The main reason why the 7 ka interval was so much profitable is probably linked to higher temperatures (Supplementary Fig. 4), which fits the western chimpanzee niche better (Fig. 6).

Niche similarity tests^55^ indicate that the western chimpanzee is not only morphologically and geographically isolated but also occupies a unique climatic space (Supplementary Fig. 8). We found that, as expected, western and eastern chimpanzees are placed towards drier, more seasonal environments, most likely including savannah conditions. However, their hydrological preferences are less well aligned. Whereas western chimpanzees live in warmer climates (Fig. 6) the eastern chimpanzee occurs in cooler habitats like the Itombwe Mountains and Virunga and Rwenzori National Parks in the Democratic Republic of Congo. In contrast to the two ‘savannah chimpanzee’, the central chimpanzee prefers more humid habitats (Fig. 6), consistent with its current geographic range encompassing lowland and swamp forests in the Congo basin. The reconstructed migration paths primarily traverse regions with high environmental similarity to modern habitats. This provides strong support for our assumption of temporal niche conservatism and suggests that our identification of ‘permeability windows’ is not an artefact of model extrapolation into unknown climatic spaces (Supplementary Fig. 10). Overall, these differences provide the ecological basis for our presumed permeability windows connecting populations through and around the Dahomey Gap, supporting the view that increased tree cover and the onset of more humid or cooler conditions may have allowed individuals of distinct population to cross the Dahomey and meet western chimpanzees at three specific time-windows (i.e., 167-160 ka, again at 100-95 ka, and at 7 ka). Although finding profitable conditions for introgression is no evidence that the gene flow actually took place then and there, and assuming the climatic preferences of common chimpanzee subspecies today stood still throughout the existence of these lineages, there is independent supporting evidence that the chimpanzees could have effectively used this time window and places to come in contact. Although the vegetation in the area around the Dahomey Gap is qualitatively similar to the current lineage’s environmental preferences, the megabiomes intersecting along the paths always have had a greater proportion of forests than the area around the Gap. Chimpanzees living in open environments exploit a larger home range and can cover longer distances^56–59^, indicating that our results are suggestive of an active selection for extensive tree cover during migration, which is a plesiomorphic character for greater apes. Moreover, although the current common chimpanzee subspecies distribution is a limited representation of their historical range^60^, they all occur in their most suitable habitats^37^, indicating that the perceived climatic niches today are a good representation of the subspecies’ historical preferences. It is important to note that our occurrence data points were derived from IUCN range polygons. While these expert-based maps may occasionally be spatially coarse and include commission errors, their impact is mitigated in our case by both the exceptional sampling effort available for chimpanzees and the macroecological scale of our analyses. IUCN maps for *Pan* are based on extensive field surveys and provide a robust approximation of broad-scale realised distributions^61,62^. In addition, the use of a 0.5° spatial resolution reduces the influence of fine-scale inaccuracies, allowing models to capture general climatic suitability rather than local occupancy patterns^63^. As such, our results should be interpreted as conservative, large-scale reconstructions of habitat suitability and connectivity.

Overall, our findings show where, when and under which climate conditions the common chimpanzee lineages, now partitioned into almost exclusive and non-overlapping habitats, were able to exchange genes in the past, providing key insights into the evolutionary history of our closest living relatives despite a near-absent fossil record.

## Materials and Methods

### Geometric morphometrics data and methods

The morphological (skull shape) dataset is composed by 49 specimens belonging to ten different Operational Taxonomic Group (OUT): *Gorilla beringei* (*n* = 2), *Gorilla gorilla* (*n* = 2), *Pan paniscus* (*n* = 2), *Pan t. ellioti* (*n* = 3), *Pan t. schweinfurthii* (*n* = 15), *Pan t. troglodytes* (*n* = 14), *Pan t. verus* (*n* = 4), *Pongo abelii* (*n* = 2), *Pongo pygmaeus* (*n* = 2), *Symphalangus syndactylus* (*n* = 2).

The CT-scans and the three-dimensional surfaces of the crania were downloaded from online repositories (i.e. Morphosource) when available. Otherwise, they were made available upon request by the Smithsonian Institution. A list of all online repositories from where CT-scans were downloaded can be found in Supplementary Information (as Appendix 1) and on Zenodo repository (doi:10.5281/zenodo.19892162). The collected surfaces and scans belong to the following institutions: Smithsonian Institute (USNM), American Museum of Natural History (AMNH), Museum of Comparative Zoology (MCZ), Royal Museum for Central Africa (RMCA), Natural History Museum London (NHMUK); Stony Brook University (SBU).

We manually sampled 56 three-dimensional anatomical landmarks, using the software Amira (version 5.4.5). The list of the collected landmarks with their description is reported in Supplementary Table 1 and Supplementary Fig. 1.

We implemented shape information using semilandmarks^64^. Semilandmarks were sampled upon a surface patch generated from the specimen closest to the consensus shape, through the K-means algorithm implemented in the R package Morpho (*kmeans* function^65^). The patch was placed upon all other surfaces and slid to secure the geometric homology^66^.

We performed a Procrustes registration and superimposition of the semilandmarks sets and then a Principal Component Analysis (PCA, Supplementary Fig. 2) to retrieve Principal Component scores.

We used *RRphylo*^67^ to compute evolutionary rates of cranial shapes among Hominoidea, using PC scores (average scores per OTU) as the shape variables. The backbone phylogeny was obtained from ref. ^68^, and then calibrated according to the split ages among greater apes following ref. ^23^ using the function *scaleTree* in RRphylo.

### Phylogenetic vector analysis of phenotypic change

To analyse the direction and the magnitude of skull shape change among *Pan troglodytes* subspecies, we used the *evo.dir* function from RRphylo^69^. This method relies on the computation of vectors of evolutionary change derived from regression coefficients (rates) associated with PC scores. These vectors describe the direction and magnitude of shape change along a given evolutionary path, from a specific node to the terminal species.

While the vector magnitude indicates the evolutionary rate, their direction can be quantified in terms of the angle they form with one another or with a reference vector (i.e., a given node along the path). Here, the most recent common ancestor (MRCA) to all *Pan troglodytes* subspecies was set as the reference. We then repeated the analysis using the most recent common ancestor of all greater apes getting, for what concerns the common chimpanzee subspecies, the same insights as with the less inclusive MRCA (Supplemetary Fig. 3).

### *rate.map* and the localisation of the shape change

We used the *rate.map* algorithm^70^ (embedded in the R package RRmorph^71^) to chart the shape change associated to the highest evolutionary rates. The *rate.map* method works by comparing a pair of species with their last common ancestor (or a species with any other node along its evolutionary path). To run *rate.map*, we first generated a three-dimensional surface of the consensus shape, which was used as a reference for shape comparison. We then compared each species on the tree to the *Pan troglodytes* subspecies MRCA. Our aim was to map the areas that underwent the most significant changes during the evolution of the given species, to identify patterns of change and characterise the morphological differentiation among the subspecies.

As final step, we used the brand-new interpolation algorithm named *interpolMesh* (RRmorph R package^71^) to transfer the rate mapping onto real surfaces to enhance the morphological comparisons.

It is important to note that our dataset does not include male individuals for NC chimpanzee, which were unavailable. To face this limitation, we repeated the analyses on a reduced dataset inclusive of females only.

### Historical biogeography data and methods

#### Catarrhine occurrences

We obtained current spatial distribution data for *Pan* species in Esri shapefile format from the IUCN Red List website (www.iucnredlist.org). The shapefile for *P. troglodytes* was further subdivided to consider its four recognised subspecies: *P. t. ellioti*, *P. t. troglodytes*, *P. t. verus*, and *P. t. schweinfurthii* (Fig.1, see supplementary material for shapefile availability). To reflect their broad geographic distribution patterns and in keeping with their phylogenetic position, we aggregated the spatial polygons of *P. t. ellioti* and *P. t. verus* to define a “western” clade, and those of *P. t. troglodytes* and *P. t. schweinfurthii* to define an “eastern” clade. Additionally, we merged the spatial polygons of all *Pan troglodytes subspecies* to represent the overall *P. troglodytes* clade spatial distribution at the species level.

#### Climatic variables

To estimate the climatic niches of *Pan troglodytes* subspecies, we used the bioclimatic variables described in ref. ^41^. Specifically, we relied on variables derived from the realistic Community Earth System Model (CESM) 1.2 paleo climate model simulation^72^ which covers the past 3 million years. The bioclimatic variables were downscaled to a spatial resolution of 0.5° × 0.5° (30 arc-min × 30 arc-min; ≈ 55 km at the equator). We excluded annual mean diurnal range (BIO2) and isothermality (BIO3) because for the CESM1.2 paleoclimate simulations, sub-daily data were not saved^73^. To avoid potential problems of multicollinearity, we first cropped the variables to the African continent, then we applied a Variance Inflation Factor (VIF) analysis with a threshold of VIF ≤ 5 using the ‘usdm’ R package^74^, retaining the following eight predictors: bio4 (temperature seasonality), bio5 (max temperature of warmest month), bio6 (min temperature of coldest Month), bio8 (mean temperature of the wettest quarter), bio9 (mean temperature of driest quarter), bio13 (precipitation of wettest month), bio14 (precipitation of driest month), and bio15 (precipitation seasonality).

#### Species distribution models (SDMs)

We trained Species Distribution Models (SDMs) to predict the suitable area of *Pan* species/subspecies and for the western and eastern clades for 1 kyr intervals in the past. For each species, we included all the species occurrences plus 10,000 background points. As occurrence data, we considered all the 0.5 ×0.5° grid cells intersecting the subspecies’ IUCN range polygons. Background points were generated by randomly sampling 10,000 points across the African mainland. Climatic data from the CESM1.2 model 3 Ma transient simulation were then extracted for both presence and background locations. For each taxon, a single SDM was calibrated by using the maximum entropy modelling algorithm (MaxEnt^40^). Model tuning was performed using the ENMeval R package version 2.0.5^75^ to optimise the balance between model fit and complexity. Specifically, we explored a range of regularisation values between 0.5 and 4, with 0.5 steps across alternative combinations of feature classes in turn: linear, linear + quadratic, hinge, linear + quadratic + hinge, linear + quadratic + hinge + product and linear + quadratic + hinge + product + threshold^75^. This procedure yielded 48 candidate models for each taxon. The best-performing model was selected based on the lowest Akaike information criterion corrected for a small sample size (AICc). To evaluate SDM predictive performance, we subsequently used a spatial block cross-validation approach, splitting the data into four spatially distinct folds of equal size. Each fold was held out in turn for validation, while the remaining folds were used for model calibration. Predictive performance was evaluated by calculating the area under the receiver operating characteristic curve (AUC^76^). Eventually, the best-fit model for each subspecies was spatially projected over 1 kyr time intervals, considering the temporal uncertainty in divergence time estimated in the *Pan* phylogeny^23^ as detailed below.

To evaluate whether SDMs calibrated with present-day climate were extrapolated into non-analogue paleoclimatic contexts, we calculated the Multivariate Environmental Similarity Surface (MESS) by using the ecospat R package^77^. Specifically, we used the present-day (0 kya) climatic layer as the calibration environment and each past time slice over the last 2 Ma ago (at 1kyr time resolutions) as the projection environment. Then, we stacked the resulting MESS maps to tally, for each grid cell, the number of slices with non-analogue conditions (i.e., negative values indicating that at least one predictor outside the calibration range) (Supplementary Fig. 10). In addition, we quantified the percentage of negative cells within polygons of each *Pan* species/subspecies. For these percentage summaries, we restricted the temporal window to the taxon-specific interval from its inferred origin to the present, according to ref. ^23^, and reported both the mean and the range across that interval (Supplementary Fig. 10).

#### RRphylogeography

After calibrating the SDMs, we used all the model projections to run the recently developed *RRphylogeography* method^41^. Specifically, *RRphylogeography* estimates the potential AOO and the zone of past contact between pairs of lineages. The geographic extent of *RRphylogeography* predictions was defined based on the merged IUCN distribution ranges of all subspecies. Specifically, we designed a global geographical extent spanning the African continent ± 20° latitudinal degrees from the equator. For each pair of subspecies, *RRphylogeography* was calibrated by incorporating the estimated divergence times and the associated uncertainty intervals between *Pan* clades as reported by refs. ^22,23^. In particular, since the split age between NC and western chimpanzee is calibrated at 250 ka^23^, we estimated the AOO of the two subspecies in the 280–220 ka interval. The split between eastern and central chimpanzee is reported within a maximum credible interval spanning from 182 to 139 ka^23^ which we followed here. The split between the western and eastern clades is reported between 633 and 544 ka, which we therefore used as the AOO sampling interval. Eventually, separation between common chimpanzee and bonobo is attested with a wide gap, between 2.10 Ma and 1.66 Ma in ref. ^23^. In ref. ^22^, the most recent common ancestor age of the clade including the bonobo, common chimpanzee and an extinct *Pan* lineage is reported at 2.03 Ma. Here, we tentatively adopted a time interval of 100 kyrs, from 1.93 and 1.83 Ma, as to include the split age between *Pan troglodytes* and *Pan paniscus* using the midpoint estimate in ref. ^23^ as an indication.

To obtain the final maps, we averaged all the resulting relative occurrence probability (RPO) maps across time, explicitly weighing them according to the divergence ages and the corresponding uncertainties.

#### Estimating Migration Pathways between *Pan troglodytes* subspecies

We estimated the most probable geographic paths connecting *Pan troglodytes* subspecies leading to the undated, geographically unexplicit introgression events recognised in genomic studies^22,78^.

To identify geographic locations connecting the origin and destination of the migration paths, we first extended the current spatial ranges of each *Pan troglodytes* subspecies by a 100 km buffer to account for their potential dispersal over currently unoccupied areas. Within this new extent, we computed the weighted barycentre of the habitat suitability map as to represent the most likely central location of the subspecies distribution. Then, for each pair of subspecies, we constructed a conductance matrix over the global geographical extent and calculated the least-cost path between the subspecies barycenters using the leastcostpath R package^79^. Conductance is proportional to habitat suitability^79^. Here, we averaged the geometric and the arithmetic means of predicted suitability values for each pair of subspecies, which is known to confer a robust consensual indicator of the habitat suitability^80^.

The entire procedure was repeated for each pairwise comparison between the western chimpanzee and the other three subspecies. To account for temporal dynamics, we estimated least-cost paths across the last 250 kyr according to the divergence times and phylogenetic relationships among *Pan* clades^23^, a period during which gene flow was detected between western-central, western-eastern, and western–ancestral central/eastern populations^22,28,78^. To find the most probable time windows for *Pan* introgressions, we first retained only those paths with costs below the 95^th^ percentile of the cost distribution per pair across all time bins. Then, we used the gridded megabiome maps published in ref. ^43^ to calculate the percentage of cells along each most probable path that were ascribed to any megabiome type (Supplementary Table 3). The same procedure was repeated over a bounding box enclosing the Dahomey Gap spatial extent (Supplementary Table 4), which is known to form an ecological barrier between *P. t. versus* the other common chimpanzee subspecies.

#### Niche overlap

To assess the similarity in environmental preferences among the *Pan troglodytes* subspecies, we compared their climatic niches using the framework described in ref. ^55^. Specifically, we employed the climatic values of the selected bioclimatic predictors associated with modern occurrences and background points to calculate the Schoener’s D metric, which quantifies the degree of niche overlap among species, ranging from low (D = 0) to perfect (D = 1). In addition, we tested whether the observed overlaps were greater than expected given the available background environments applying the niche similarity test with 10,000 permutations, conducted in both directions (species A against species B’s background, and vice versa). The analysis of niche similarity allows us to quantify the ecological divergence between subspecies, which is key to figure out how likely is (and under which conditions) that any pair of them came in contact. Schoener’s D calculations and niche similarity test were conducted using the ecospat R package^77^.

### Statistics and reproducibility

Statistical analysis was done with R Studio Software version 2025.09.2 based on R 4.5.0. The analysis of shape was mainly conducted through the packages Morpho and Rvcg^65^, while the evolutionary rate computation and the evolutionary trajectories were computed via RRphylo^67,69^. Digital models of the skull were downloaded from public repositories (i.e. Morphosource) or available upon request (see Appendix 1, Supplementary Information). We decided to include only the specimens that were born in nature.

The estimations of Area of Origins (AOO) were performed with the new RRgeo R package (https://cran.r-project.org/web/packages/RRgeo/index.html). *Pan* models were calibrated by using the functionality embedded in the biomod2 R package version 4.2-6^81^. Niche overlap estimations and niche similarity test followed the approach described in Broenimann et al. (2012). To run these tests, we used the ecospat R package^77^.

Figures and plots were prepared using the R package ggplot2^82^ and the Photoshop suite.

### Reporting summary

Further information on research design is available in the Nature Portfolio Reporting Summary linked to this article.

## Supplementary information

Supplementary Material

Lasing Report

Reporting Summary

## Data Availability

Data sources and input data to replicate the analyses are available on Zenodo^83^ with the following https://doi.org/10.5281/zenodo.19892162.

## Code availability

Software and R codes to replicate the analyses are available on Zenodo^83^ with the following https://doi.org/10.5281/zenodo.19892162.
