# Supplementary material for: Evidence for multiple past introgressions among chimpanzee subspecies during warming periods
Source: Commun Biol. 2026 Jun 5;9:1188. doi: 10.1038/s42003-026-10437-z (PMC13558573; doi:10.1038/s42003-026-10437-z)
Supplement: Supplementary file 1 [file 42003_2026_10437_MOESM1_ESM.pdf]

Supplementary Information

Evidence for multiple past introgressions among chimpanzee subspecies during warming periods

Mondanaro Alessandro[1], Melchionna Marina[1], Serio Carmela[1], Girardi Giorgia[1], Timmermann Axel[2], Zeller Elke[3], Castiglione Silvia[1], Di Costanzo Alessia[1], Morvillo Linda[1], Esposito Antonella[1], de Durante Alessandra[4], Di Febbraro Mirko[4], Raia Pasquale[1]*

[1] These authors contributed equally

*author for correspondence

[1]DiSTAR, University of Naples Federico II; 80126 Naples, Italy.

[2]IBS Center for Climate Physics, Institute for Basic Science, Busan, South Korea.

[3] Department of Geosciences, University of Arizona, Tucson, USA.

[4]EnviXLab, Department of Biosciences and Territory, University of Molise; 86090 Pesche (Isernia), Italy.

*author for correspondence: Prof. Pasquale Raia pasquale.raia@unina.it

Supplementary Methods

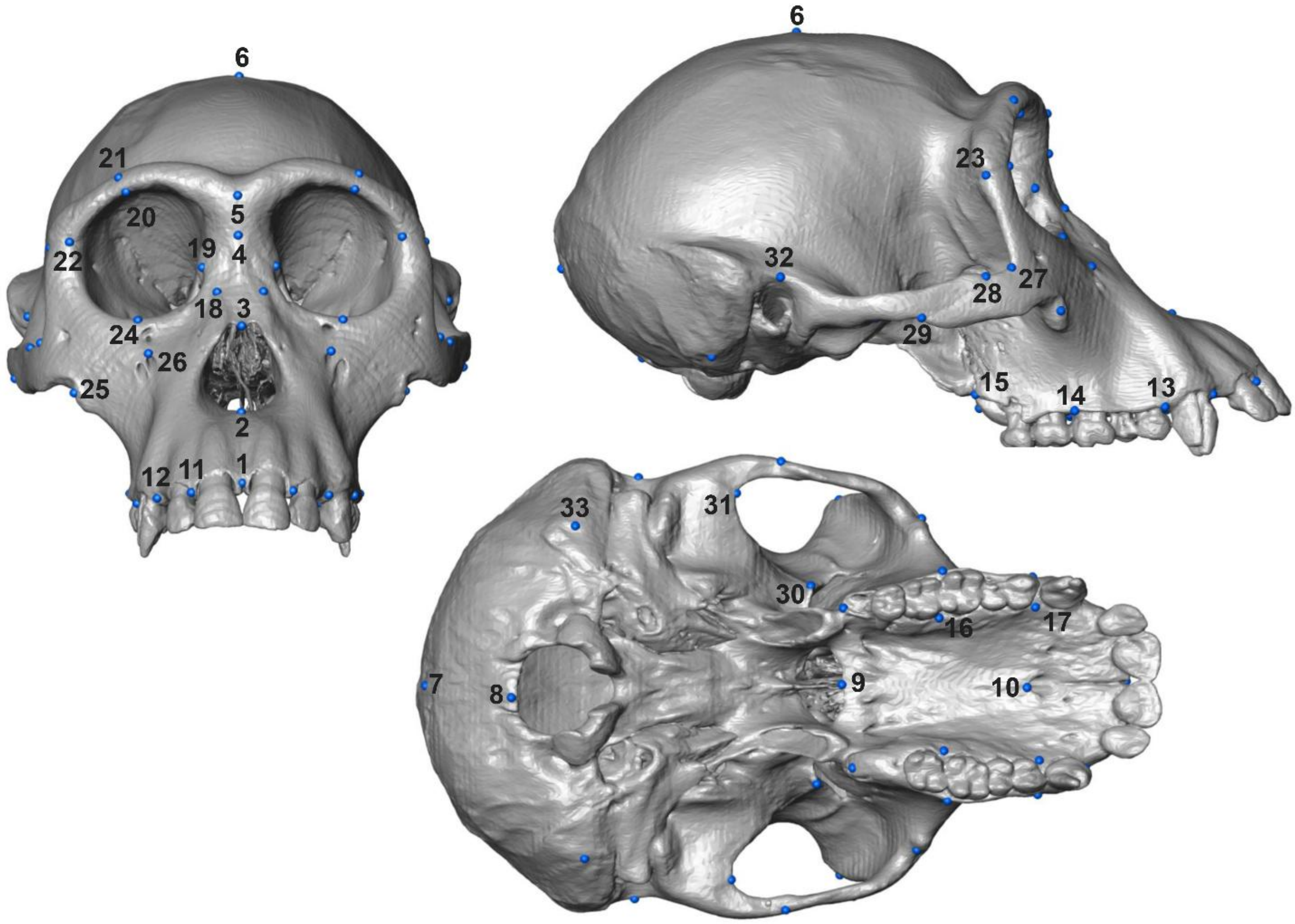

Supplementary Fig. 1. Landmark configuration used for the Geometric Morphometric analyses. Only the right landmarks are indicated. The full landmarks list and description are reported in Supplementary Table 1.

Supplementary Table 1. Landmarks description.

	Midline	
1	Prosthion	Anteroinferior point on projection of premaxilla between central incisors
2	Nasospinale	Inferiormost midline point on piriform aperture
3	Rhinion	Most anterior point in midline on nasals.
4	Nasion	Fronto-nasal suture in midline
5	Glabella	As viewed in Frankfurt horizontal
6	Bregma	Junction of coronal/sagittal sutures, on sagittal crest if necessary
7	Opistocranion	Most posterior median point on the occipital bone, instrumentally determined as the greatest chord length from the glabella
8	Opisthion	Posterior most point of foramen magnum
9	Staphylion	Midline point on palate on line tangent to anterior most points on choanae
10	Incisivion	Midline point at the anterior most point of the maxilla (=posterior end of incisive foramen), extrapolated if broken or asymmetrical

	Bilateral (R-L)	
11-34	Prosthion2	Antero-inferiormost point on premaxilla, equivalent to prosthion, but between central and lateral incisors
12-35	Pre-canine	Anterior most point of the alveolar canine
13-36	Mesial M1-2 contact	Projected (laterally) onto alveolar margin (mesial)
14-37	Mesial P3	Most mesial point on P3 alveolus, projected onto alveolar margin
15-38	Maxillary tuberosity	Lower part of the infratemporal surface of maxilla
16-39	Buccal P3	Most buccal point on P3 alveolus, projected onto alveolar margin
17-40	Buccal M1-2 contact	Projected (laterally) onto alveolar margin (buccal)
18-41	Premax-max superior	Where premaxillo-maxillary suture meets nasal bone, or aperture if it doesn't continue to nasal bone.
19-42	Dacryon	Junction of frontal, lacrimal and maxilla
20-43	Mid-torus inferior	Point on inferior margin of supraorbital torus (superior margin of orbit) roughly at middle of orbit
21-44	Mid-torus superior	Superior to MTI on superior most point of supraorbital torus when viewed in Frankfurt horizontal
22-45	Frontomalare orbitale	Where the frontozygomatic suture crosses the inner orbital rim
23-46	Frontomalare temporale	Where the frontozygomatic suture crosses the lateral edge of zygoma
24-47	Zygo-max superior	Anterosuperior point of zygomaticomaxillary suture (taken at orbit rim)
25-48	Zygo-max inferior	Anteroinferior point of zygomaticomaxillary suture, in antero-lateral view
26-49	Infraorbital foramen	Tip
27-50	Zygo-arch	Anterior most point of the zygomatic arch
28-51	Zygo-temp superior	Superior point of zygomatico-temporal suture on lateral face of zygomatic arch
29-52	Zygo-temp inferior	Inferolateral point of zygomaticotemporal suture on lateral face of zygomatic arch
30-53	Infratemporal crest	Tip
31-54	Lateralglenoid	Tip
32-55	Porion	(In Frankfurt horizontal–defines) top of auditory meatus
33-56	Mastoid process	Lower part of the mastoid process

Supplementary Results

The shape and the direction of the morphological differentiation among *Pan* subspecies: *evo.dir* and *rate.map*.

To investigate the morphological variation among *Pan* subspecies (i.e. *P. t. ellioti*, *P. t. schweinfurthii*, *P. t. troglodytes*, *P. t. verus*) under a phylogenetically explicit context, it is necessary to consider the wider context of the Hominoidea clade. Therefore, the analysis of shapes also includes *Gorilla beringei*, *Gorilla gorilla*, *Pan paniscus*, *Pongo abelii*, *Pongo pygmaeus*, *Symphalangus syndactylus*. The presence of *S. syndactylus* as an outgroup is necessary to polarize the morphological change.

We performed a Principal Component Analysis (PCA) to study the morphospace and check for the relative position of our specimens, both male, female and unspecified individuals (Supplementary Fig. 2). The first Principal Component (PC1) accounts for 41.24% of the total variance. At the positive end of this axis, *Pan paniscus* can be observed, followed by the *Pan* subspecies, which largely overlap with one another. At the negative end, we find the male specimens of *G. beringei* and *P. abelii*. The second Principal Component (PC2) explains 14.35% of the total variance and separates *S. syndactylus* (negative values) from the great apes (positive values).

Once the morphological vectors (i.e., PC scores) for Hominoidea species were obtained, we applied the *RRphylo* method (Castiglione et al., 2018) to compute evolutionary rates, which represents the magnitude of shape change over time. As *RRphylo* is a phylogenetic comparative regression method, it produces vectors of regression coefficients, describing the directions, alongside the magnitude, of shape change. With *evo.dir* function included in RRphylo package, we computed the angles between each *Pan* subspecies and its most recent common ancestor (MRCA). The angle of *P. t. verus* to the MRCA is the highest (149.6 degrees, p value < 0.01, Supplementary Table 2) as for the magnitude. Given the limited availability of specimens from both sexes for *P. t. ellioti*, we replicated the analysis using only female individuals. The overall pattern remained consistent, with *P. t. verus* still exhibiting the widest angle (and magnitude) between the MRCA (154.2 degrees, p value < 0.01; Supplementary Table 2).

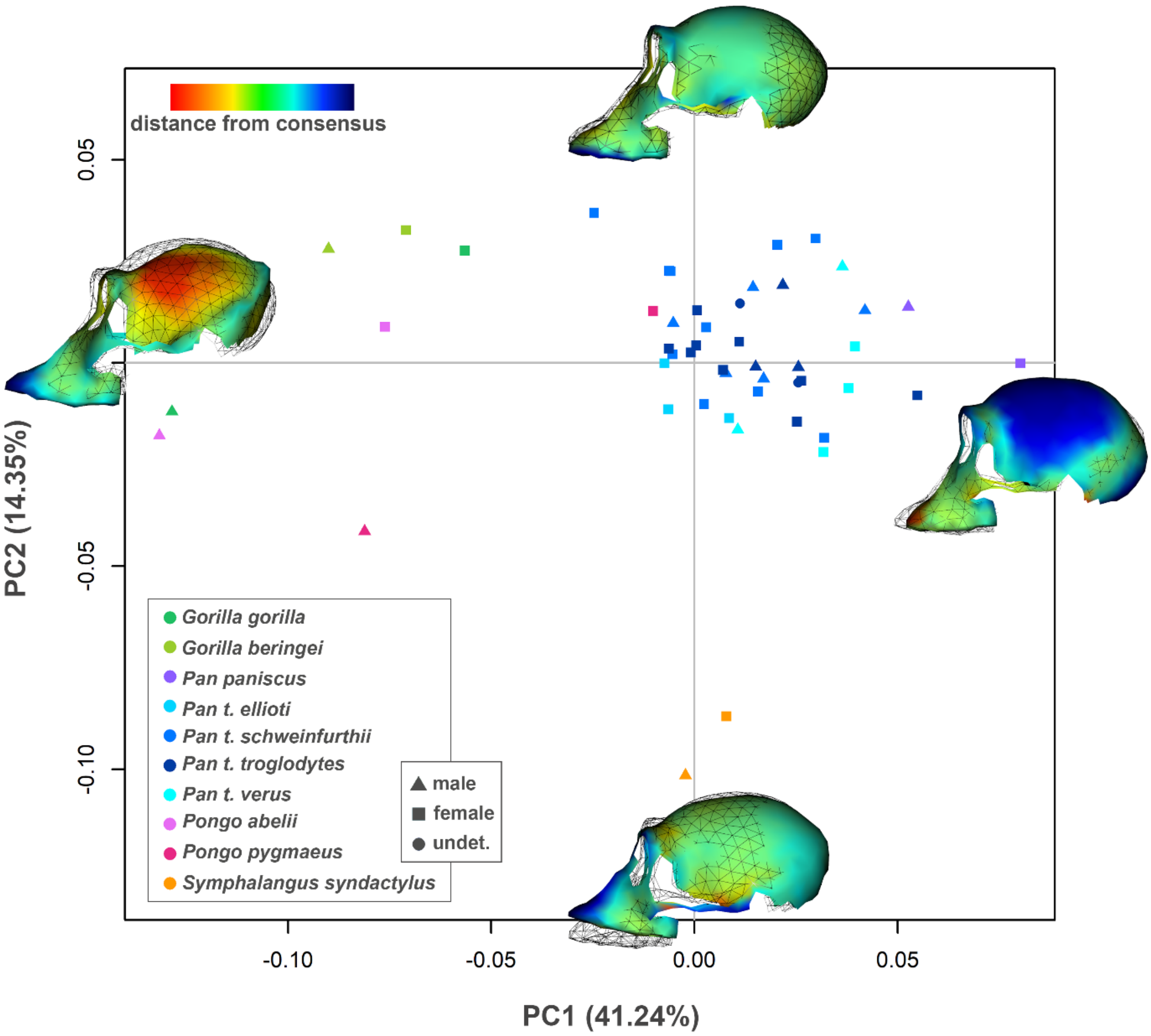

Supplementary Fig. 2. Principal Component Analysis performed on the Hominoidea cranial shape. The plot shows the occupation of the morphospace related to PC1 and PC2. The cranial shapes are reconstruction based on the extreme values of the axes and the colour gradient (from red to blue) indicate the distance with the mean shape which is the superimposed grid.

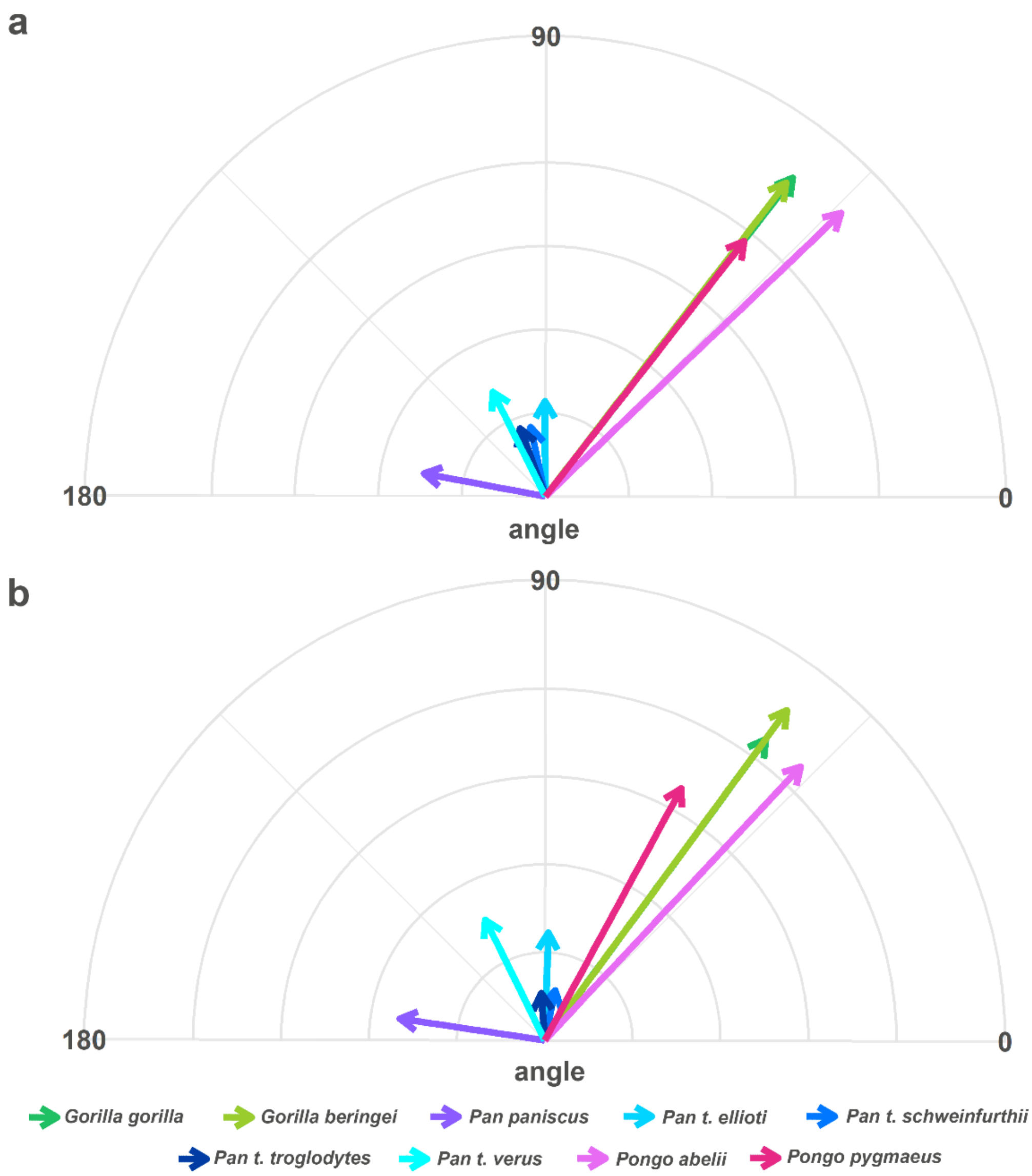

Supplementary Fig. 3. Direction, magnitude and localization of the morphological change among Hominoidea (*Symphalangus* excluded). The multivariate angles were computed against each subspecies and their MRCA. Vector length is proportional to actual vector size, which represents the evolutionary rate.

Supplementary Table 2. Multivariate angle of evolutionary rates between the *Pan* subspecies and their MRCA. The columns indicate with "all" refers to the analysis with all specimens, beside their sex attribution. The columns with "F" refer to the analysis where only the female individuals were included.

	**angle**		**vector size**		**p value**	
	**all**	**F**	**all**	**F**	**all**	**F**
Pan t. ellioti	114.737	122.279	0.033	0.037	0	0
Pan t. schweinfurthii	93.086	57.004	0.026	0.033	< 0.01	0
Pan t. troglodytes	104.923	67.195	0.024	0.023	0.01	0.02
Pan t. verus	149.565	154.223	0.043	0.052	0	< 0.01

The magnitude of the vectors represents scalar values and does not provide explicit information about the anatomical changes occurring during clade evolution. Similarly, the direction of the vectors does not offer insight into the specific anatomical regions involved. To overcome this issue, we used the *rate.map* algorithm, implemented in the R package RRmorph (Melchionna et al., 2024), to visualize the influence of high evolutionary rates directly on cranial shape variation.

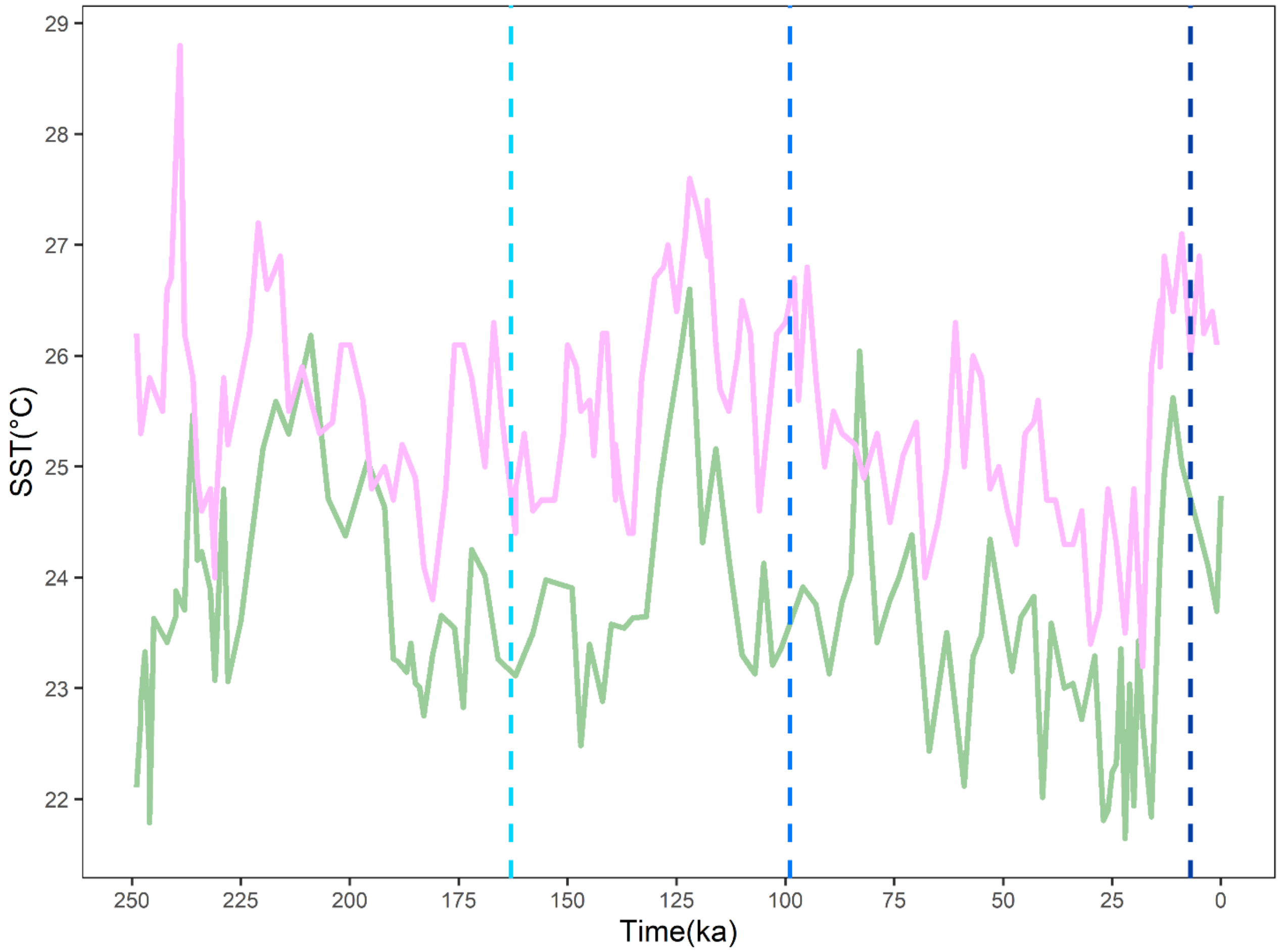

Supplementary Fig. 4. Temperature time series from Sea Surface Temperature reconstruction at core 1105 (Nürnberg et al., 2000) green line, and core 1112 (Nürnberg et al., 2000), pink line. The vertical dashed lines represent least cost moments in time for the contact between western and NC common chimpanzees (light blue at 163 ka); between western and eastern chimpanzee (blue at 99 ka); and between western and central chimpanzee (dark blue at 7 ka).

Species Distribution Models (SDMs)

SDMs demonstrated excellent predictive performance. By averaging the AUC scores of each separately modelled *Pan* species and subspecies, we obtained an AUC value of 0.960 (SD = 0.025). For the models focusing specifically on the "western" and "eastern" *Pan* clades, the average AUC remained high at 0.909 (SD = 0.011). Eventually, models calibrated using all *P. troglodytes* subspecies occurrences achieved an AUC of 0.845. Regarding the transferability of models in the past, MESS results show that extrapolation was minimal overall, weak and spatially limited only for *P. t. troglodytes* and negligible for the remaining taxa (Supplementary Fig. 10) supporting the robustness of paleo-projections and the reliability of downstream analyses based on them.

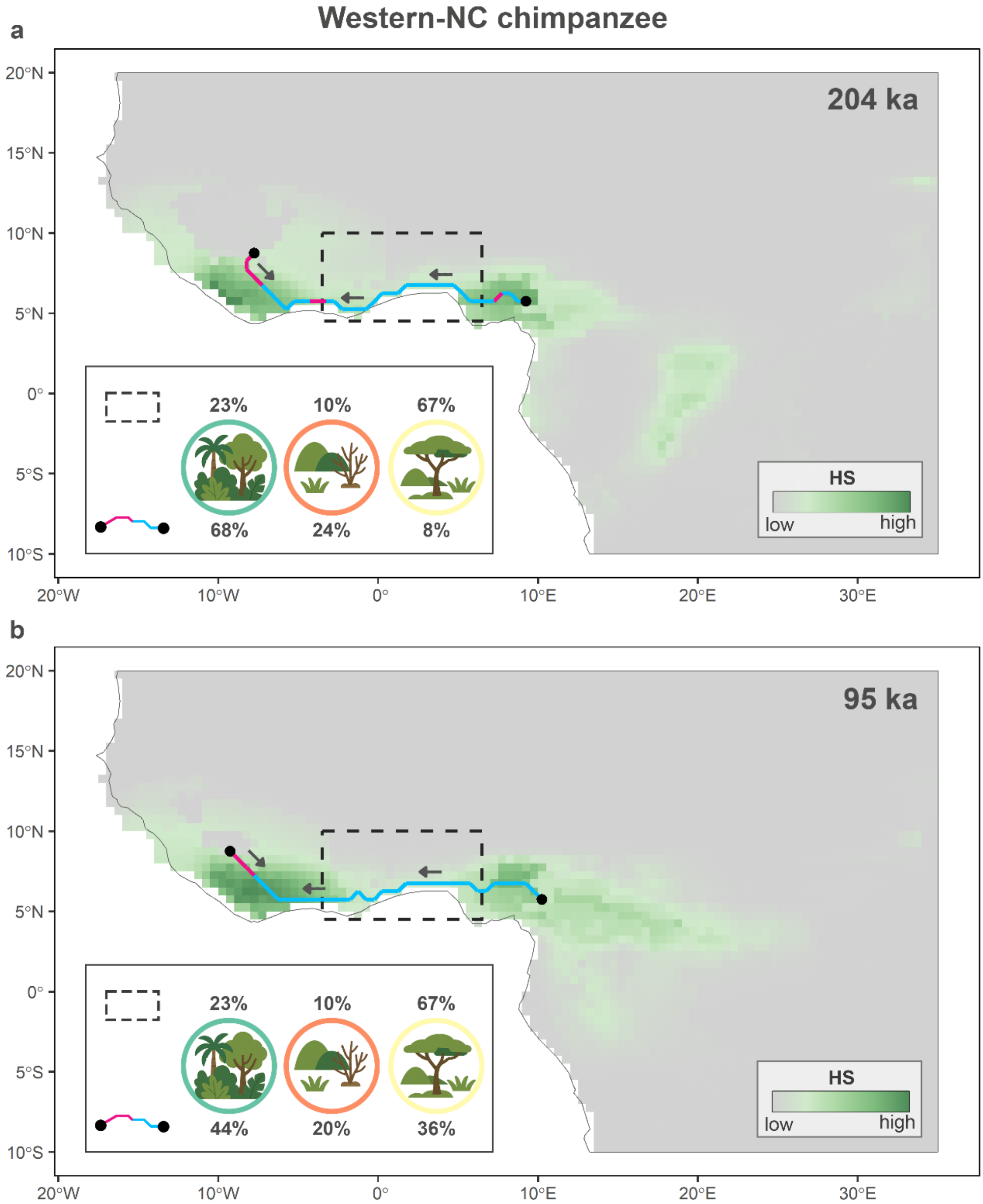

Supplementary Fig. 5. Combined habitat suitability (HS) maps for western-NC chimpanzees, depicted at the moments of the highest probability of contact between the two of individual populations. Segments along the path are coloured according to the subspecies possessing the higher habitat suitability value of the pair (deep pink refers to western chimpanzee, light blue to NC chimpanzee). The dashed bounding box indicates an approximation of the Dahomey Gap spatial range in the past. Percentage of megabiome coverage within the Dahomey Gap and along the pathways are reported.

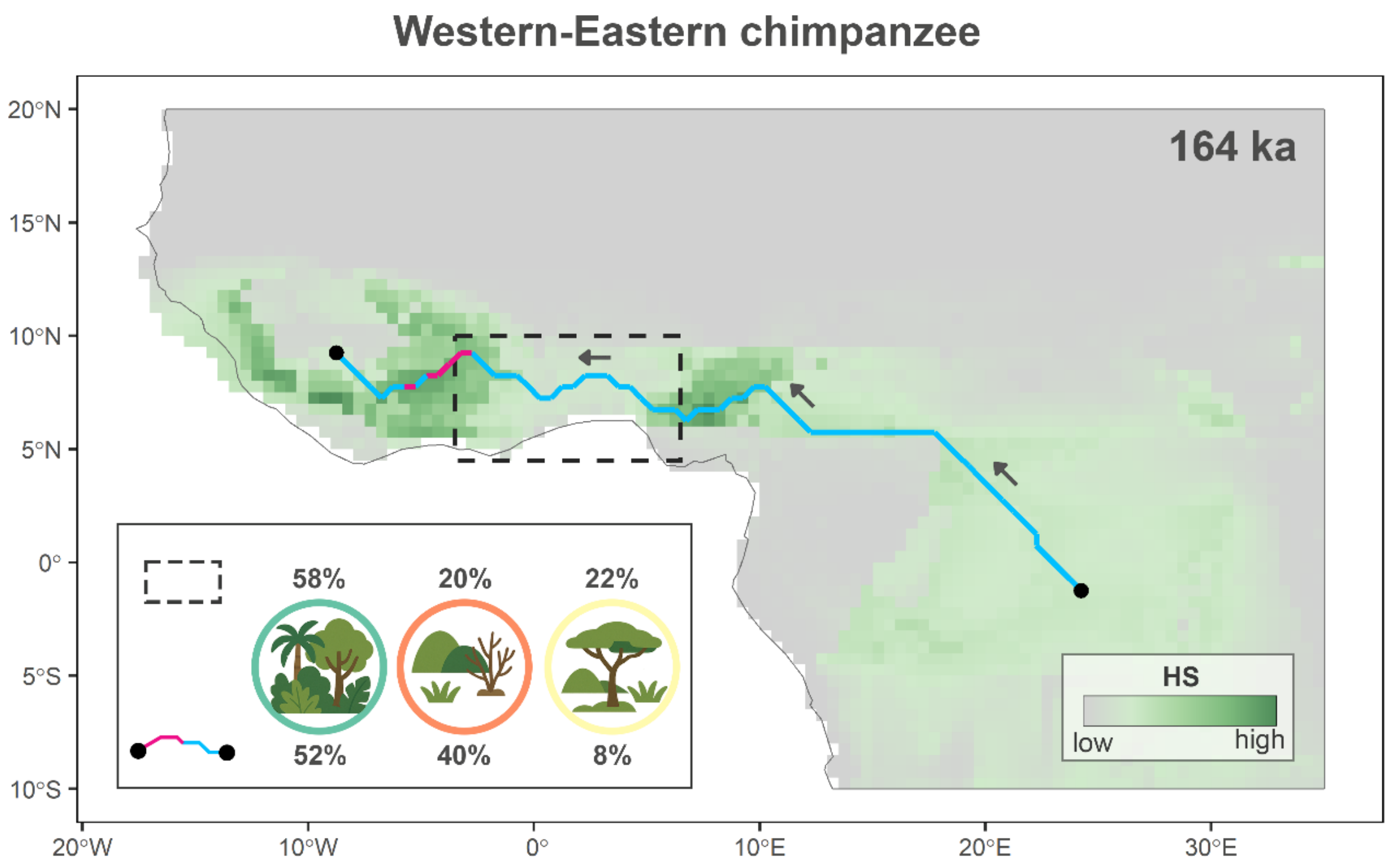

Supplementary Fig. 6. Combined habitat suitability maps (HS) for western-eastern chimpanzees, depicted at the moment of the highest probability of contact between the two of individual populations. Segments along the path are coloured according to the subspecies possessing the higher habitat suitability value of the pair (deep pink refers to western chimpanzee, light blue to eastern chimpanzee). The dashed bounding box indicates an approximation of the Dahomey Gap spatial range in the past. Percentage of megabiome coverage within the Dahomey Gap and along the pathways are reported.

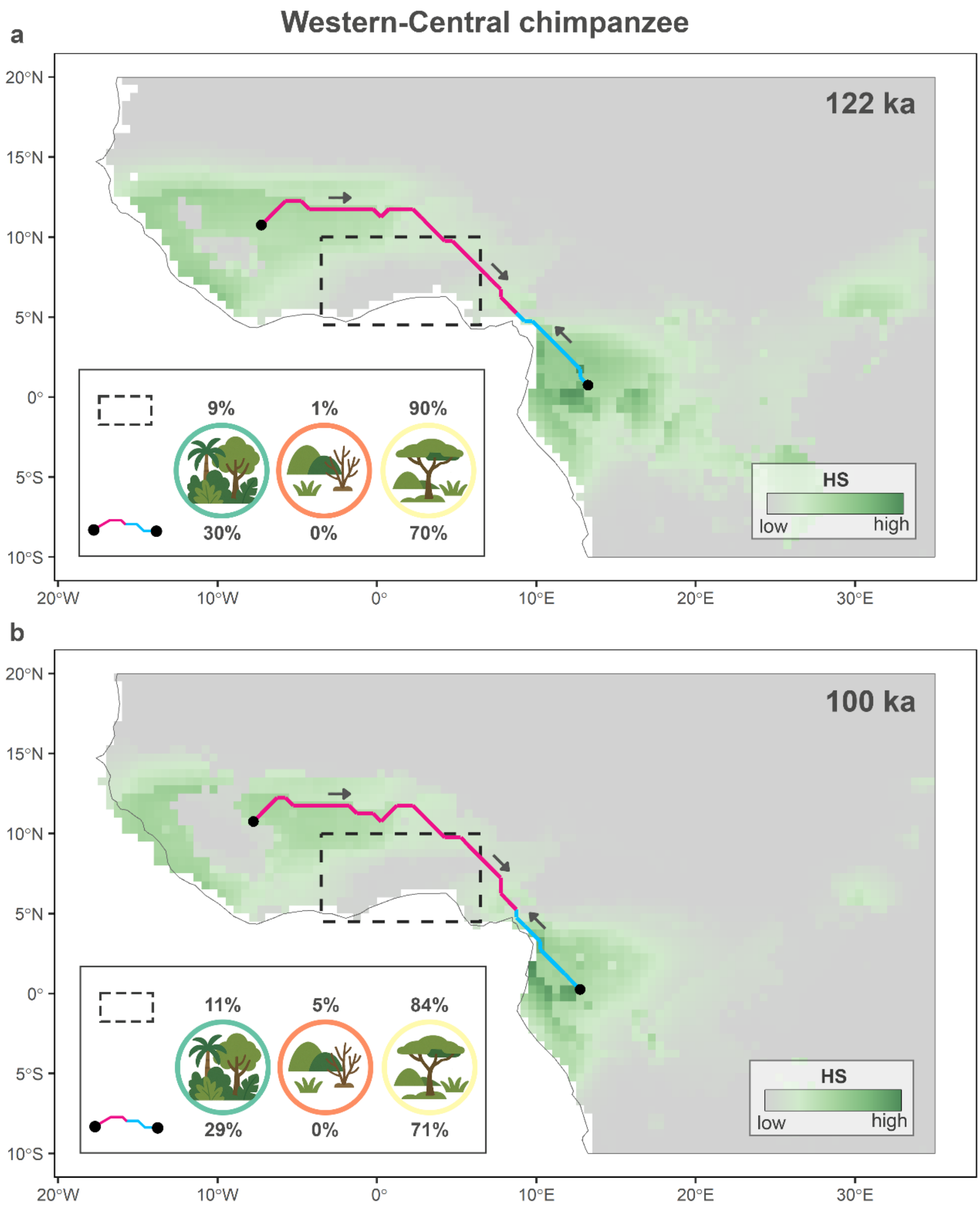

Supplementary Fig. 7. Combined habitat suitability maps (HS) for western-central chimpanzees, depicted at the moments of the highest probability of contact between the two of individual populations. Segments along the path are coloured according to the subspecies possessing the higher habitat suitability value of the pair (deep pink refers to western chimpanzee, light blue to central chimpanzee). The dashed bounding box indicates an approximation of the Dahomey Gap spatial range in the past. Percentage of megabiome coverage within the Dahomey Gap and along the pathways are reported.

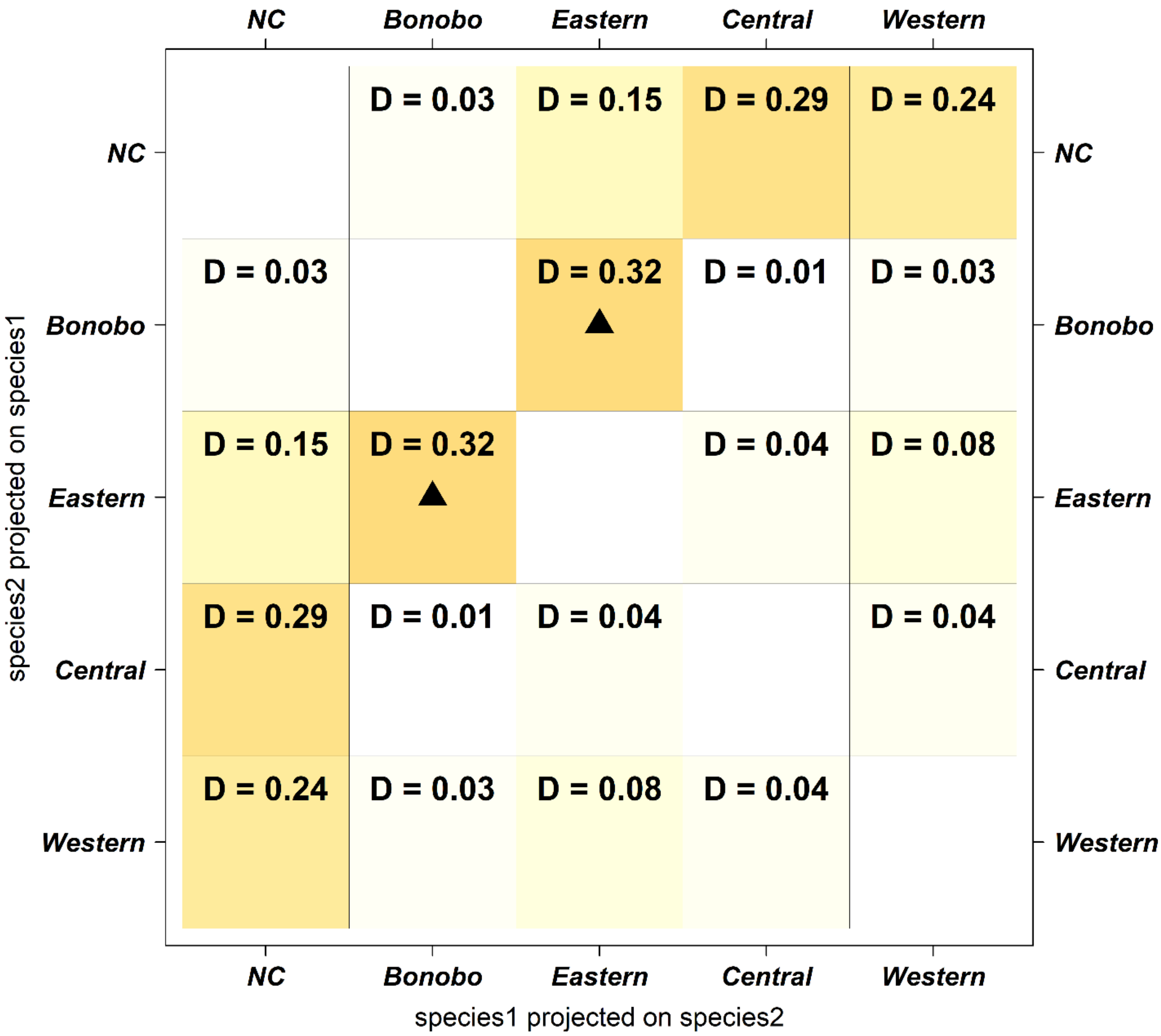

Supplementary Fig. 8. Niche similarity test based on 10,000 iterations. Boxes represent pairwise niche overlap comparisons, with the yellow–orange colour scale indicating the magnitude of Schoener's D values. Black triangles are shown only for comparisons that are statistically significant relative to random expectations.

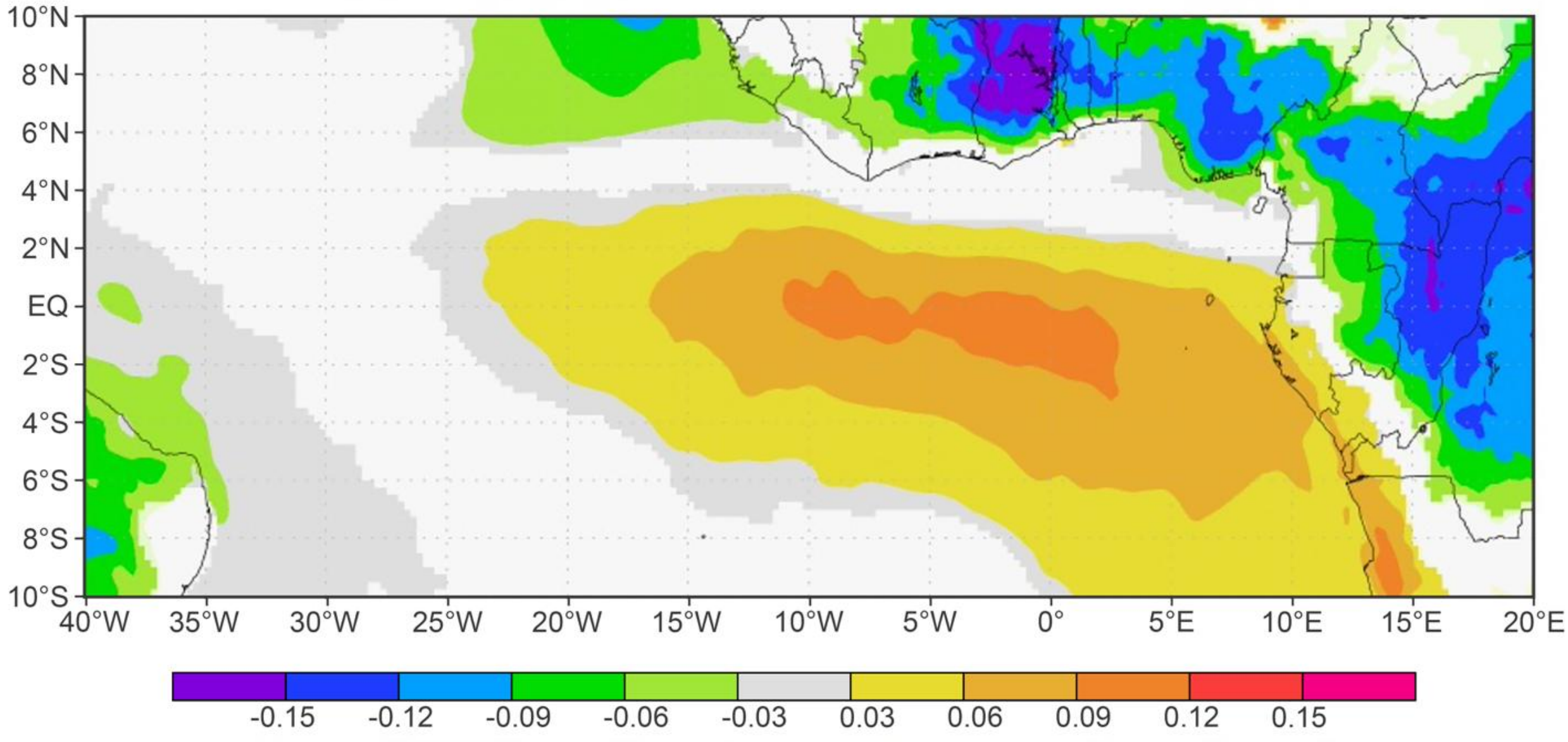

Supplementary Fig. 9. Regression between monthly, detrended precipitation anomalies averaged over the Dahomey gap (3W-3E, 6N-9N) with tropical Atlantic sea surface temperature anomalies. The Unit is K/(mm/year), and the data is from the ERA5 reanalysis. Increased rainfall over the Dahomey gap region occurs during Atlantic Niño events.

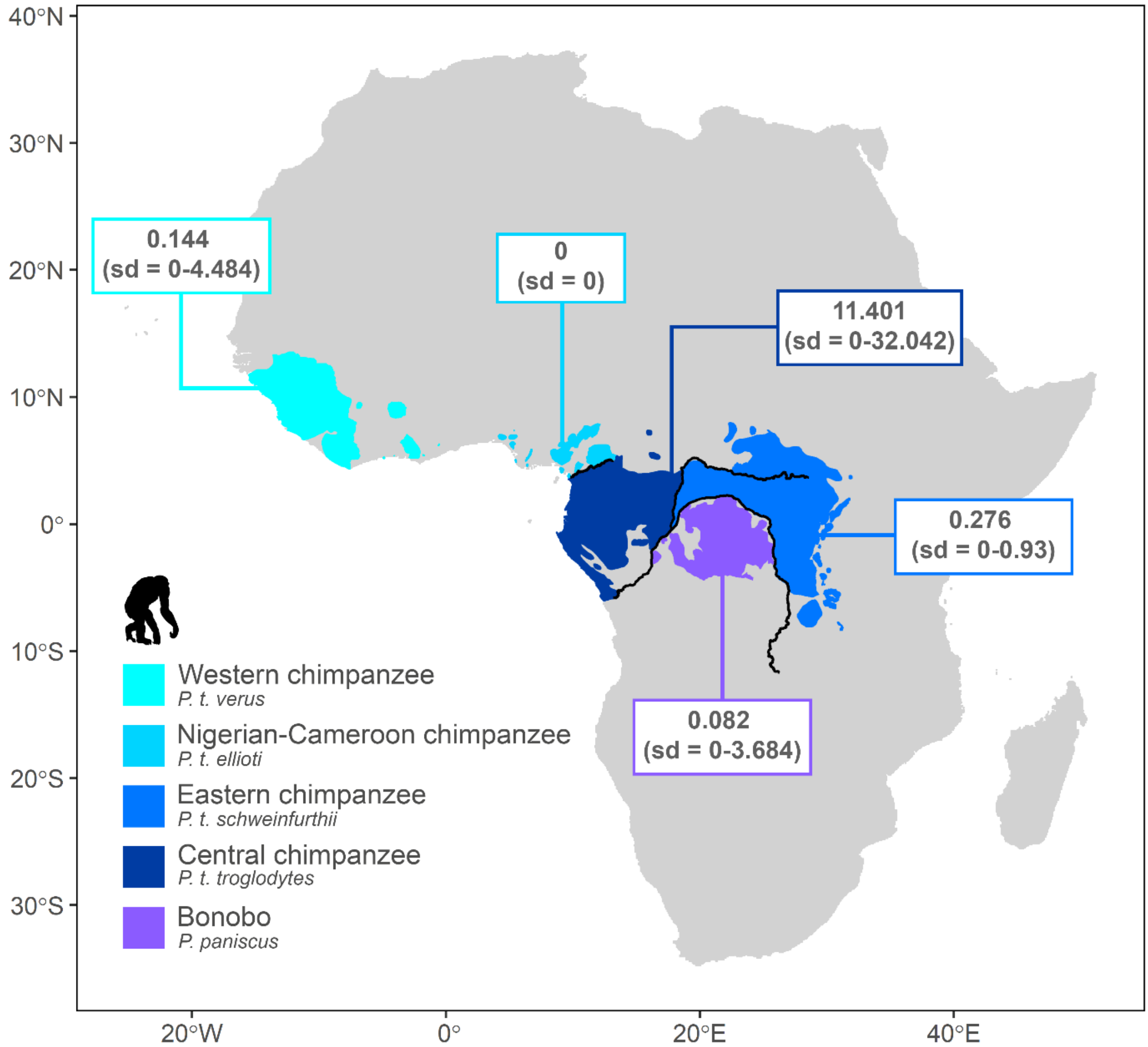

Supplementary Fig. 10. Multivariate Environmental Similarity Surfaces (MESS) calculated by using the present-day climatic layer as the calibration environment and each past time slice over the last 2 Ma as the projection environment. For each spatial polygons of *Pan* genus, we reported the mean and the standard deviation (sd) range of the number of time slices with non-analogue conditions.

Supplementary Table 3. Percentage of megabiome coverage within and surrounding the Dahomey Gap during the most probable *Pan* migration time windows.

Megabiome	Biome coverage (%)	Time (ka)	Reference
Grassland and dry shrubland	1.23	0	present
Savanna and dry woodland	81.60	0	present
Tropical forest	17.18	0	present
Grassland and dry shrubland	1.81	7	*P. t. troglodytes* peak
Savanna and dry woodland	87.35	7	*P. t. troglodytes* peak
Tropical forest	10.84	7	*P. t. troglodytes* peak
Grassland and dry shrubland	86.44	22	Last Glacial Maximum
Savanna and dry woodland	6.78	22	Last Glacial Maximum
Tropical forest	6.78	22	Last Glacial Maximum
Grassland and dry shrubland	29.07	95	*P. t. ellioti* peak
Savanna and dry woodland	55.81	95	*P. t. ellioti* peak
Tropical forest	15.12	95	*P. t. ellioti* peak
Grassland and dry shrubland	5.23	99	*P. t. schweinfurthii* peak
Savanna and dry woodland	83.72	99	*P. t. schweinfurthii* peak
Tropical forest	11.05	99	*P. t. schweinfurthii* peak
Grassland and dry shrubland	4.62	100	*P. t. troglodytes* peak
Savanna and dry woodland	84.39	100	*P. t. troglodytes* peak
Tropical forest	10.98	100	*P. t. troglodytes* peak
Grassland and dry shrubland	0.62	122	*P. t. troglodytes* peak
Savanna and dry woodland	90.06	122	*P. t. troglodytes* peak
Tropical forest	9.32	122	*P. t. troglodytes* peak
Grassland and dry shrubland	56.46	163	*P. t. ellioti* peak
Savanna and dry woodland	21.77	163	*P. t. ellioti* peak
Tropical forest	21.77	163	*P. t. ellioti* peak
Grassland and dry shrubland	57.63	164	*P. t. schweinfurthii* peak
Savanna and dry woodland	20.34	164	*P. t. schweinfurthii* peak
Tropical forest	22.03	164	*P. t. schweinfurthii* peak
Grassland and dry shrubland	10.47	204	*P. t. ellioti* peak
Savanna and dry woodland	66.86	204	*P. t. ellioti* peak
Tropical forest	22.67	204	*P. t. ellioti* peak
Grassland and dry shrubland	52.57	227	*P. t. ellioti* peak
Savanna and dry woodland	23.43	227	*P. t. ellioti* peak
Tropical forest	24.00	227	*P. t. ellioti* peak

Supplementary Table 4. Percentage of megabiome coverage along the pathways during the most probable *Pan* migration time windows.

Megabiome	Biome coverage (%)	Reference	Time (ka)
Savanna and dry woodland	65.91	*P. t. troglodytes* peak	7
Tropical forest	34.09	*P. t. troglodytes* peak	7
Grassland and dry shrubland	20.51	*P. t. ellioti* peak	95
Savanna and dry woodland	35.90	*P. t. ellioti* peak	95
Tropical forest	43.59	*P. t. ellioti* peak	95
Grassland and dry shrubland	1.61	*P. t. schweinfurthii* peak	99
Savanna and dry woodland	69.35	*P. t. schweinfurthii* peak	99
Tropical forest	29.03	*P. t. schweinfurthii* peak	99
Savanna and dry woodland	71.11	*P. t. troglodytes* peak	100
Tropical forest	28.89	*P. t. troglodytes* peak	100
Savanna and dry woodland	69.77	*P. t. troglodytes* peak	122
Tropical forest	30.23	*P. t. troglodytes* peak	122
Grassland and dry shrubland	42.50	*P. t. ellioti* peak	163
Savanna and dry woodland	10.00	*P. t. ellioti* peak	163
Tropical forest	47.50	*P. t. ellioti* peak	163
Grassland and dry shrubland	40.30	*P. t. schweinfurthii* peak	164
Savanna and dry woodland	7.46	*P. t. schweinfurthii* peak	164
Tropical forest	52.24	*P. t. schweinfurthii* peak	164
Grassland and dry shrubland	8.11	*P. t. ellioti* peak	204
Savanna and dry woodland	24.32	*P. t. ellioti* peak	204
Tropical forest	67.57	*P. t. ellioti* peak	204
Grassland and dry shrubland	27.03	*P. t. ellioti* peak	227
Savanna and dry woodland	2.70	*P. t. ellioti* peak	227
Tropical forest	70.27	*P. t. ellioti* peak	227

Appendix 1. List of specimens used for the morphological analyses. Museum abbreviation: Smithsonian Institute (USNM); American Museum of Natural History (AMNH); Museum of Comparative Zoology (Harvard) (MCZ); Royal Museum for Central Africa (RMCA); Natural History Museum London (NHMUK); Stony Brook University - Anatomical Sciences Collection (SBU).

Species	Id sample	Sex	Istitution	Source
Gorilla beringei	USNM545029	F	USNM	available upon request
Gorilla beringei	USNM397351	M	USNM	available upon request
Gorilla gorilla	USNM252577	F	USNM	available upon request
Gorilla gorilla	USNM174712	M	USNM	available upon request
Pan paniscus	AMNH-86857	F	AMNH	available on Morphosource
Pan paniscus	MCZ-38020	M	MCZ	available on Morphosource
Pan troglodytes ellioti	MCZ-23167	F	MCZ	available on Morphosource
Pan troglodytes ellioti	MCZ-26847	F	MCZ	available on Morphosource
Pan troglodytes ellioti	MCZ-26849	F	MCZ	available on Morphosource
Pan troglodytes schweinfurthii	RMCA-83006M31	F	RMCA	available on Morphosource
Pan troglodytes schweinfurthii	RMCA-83006M32	F	RMCA	available on Morphosource
Pan troglodytes schweinfurthii	RMCA-83006M33	F	RMCA	available on Morphosource
Pan troglodytes schweinfurthii	RMCA-83006M34	F	RMCA	available on Morphosource
Pan troglodytes schweinfurthii	RMCA-83006M35	F	RMCA	available on Morphosource
Pan troglodytes schweinfurthii	RMCA-83006M39	F	RMCA	available on Morphosource
Pan troglodytes schweinfurthii	RMCA-91060M405	F	RMCA	available on Morphosource
Pan troglodytes schweinfurthii	RMCA-91060M412	F	RMCA	available on Morphosource
Pan troglodytes schweinfurthii	RMCA-91060M413	F	RMCA	available on Morphosource
Pan troglodytes schweinfurthii	USNM236971	F	USNM	available upon request
Pan troglodytes schweinfurthii	RMCA-83006M13	M	RMCA	available on Morphosource
Pan troglodytes schweinfurthii	RMCA-83006M14	M	RMCA	available on Morphosource
Pan troglodytes schweinfurthii	RMCA-83006M15	M	RMCA	available upon request
Pan troglodytes schweinfurthii	RMCA-83006M19	M	RMCA	available upon request
Pan troglodytes schweinfurthii	RMCA-91060M417	M	RMCA	available upon request
Pan troglodytes troglodytes	MCZ-17702	F	MCZ	available upon request
Pan troglodytes troglodytes	USNM174699	F	USNM	available upon request
Pan troglodytes troglodytes	USNM174700	F	USNM	available upon request
Pan troglodytes troglodytes	USNM174701	F	USNM	available upon request
Pan troglodytes troglodytes	USNM174707	F	USNM	available upon request
Pan troglodytes troglodytes	USNM176229	F	USNM	available upon request
Pan troglodytes troglodytes	USNM220062	F	USNM	available upon request
Pan troglodytes troglodytes	USNM220063	F	USNM	available upon request
Pan troglodytes troglodytes	USNM599173	F	USNM	available upon request
Pan troglodytes troglodytes	USNM176228	M	USNM	available upon request
Pan troglodytes troglodytes	USNM220327	M	USNM	available upon request
Pan troglodytes troglodytes	USNM599172	M	USNM	available upon request
Pan troglodytes troglodytes	USNM176226	U	USNM	available upon request
Pan troglodytes troglodytes	USNM176238	U	USNM	available upon request

Pan troglodytes verus	RS94-4	F	SBU	available on Morphosource
Pan troglodytes verus	USNM477333	F	USNM	available upon request
Pan troglodytes verus	USNM481803	F	USNM	available upon request
Pan troglodytes verus	AMNH-89406	M	AMNH	available upon request
Pan troglodytes verus	USNM481804	M	USNM	available upon request
Pongo abelii	USNM143597	F	USNM	available upon request
Pongo abelii	USNM267325	M	USNM	available upon request
Pongo pygmaeus	MCZ-37519	F	MCZ	available on Morphosource
Pongo pygmaeus	NHMUK-1986-1097	M	NHMUK	available on Morphosource
Symphalangus syndactylus	USNM114497	F	USNM	available upon request
Symphalangus syndactylus	USNM283563	M	USNM	available upon request

References

Castiglione, S., Tesone, G., Piccolo, M., Melchionna, M., Mondanaro, A., Serio, C., Di Febbraro, M., & Raia, P. (2018). A new method for testing evolutionary rate variation and shifts in phenotypic evolution. *Methods in Ecology and Evolution*, *9*(4), 974–983. https://doi.org/10.1111/2041-210X.12954

Melchionna, M., Castiglione, S., Girardi, G., Serio, C., Esposito, A., Mondanaro, A., Profico, A., Sansalone, G., & Raia, P. (2024). RRmorph—a new R package to map phenotypic evolutionary rates and patterns on 3D meshes. *Communications Biology*, *7*(1), 1009. https://doi.org/10.1038/s42003-024-06710-8

Nürnberg, D., Müller, A., & Schneider, R. R. (2000). Paleo-sea surface temperature calculations in the equatorial east Atlantic from Mg/Ca ratios in planktic foraminifera: A comparison to sea surface temperature estimates from U 37 K′, oxygen isotopes, and foraminiferal transfer function. *Paleoceanography*, *15*(1), 124–134. https://doi.org/10.1029/1999PA000370
